# FAM64A promotes HNSCC tumorigenesis by mediating transcriptional autoregulation of FOXM1

**DOI:** 10.1038/s41368-022-00174-4

**Published:** 2022-05-10

**Authors:** Xinyuan Zhao, Huan Chen, Yu Qiu, Li Cui

**Affiliations:** 1 Stomatological Hospital, Southern Medical University, Guangzhou, China; 2 Department of Oral and Maxillofacial Surgery, the First Affiliated Hospital, Fujian Medical University, Fuzhou, China; 3 School of Dentistry, University of California, Los Angeles, Los Angeles, CA USA

**Keywords:** Mechanisms of disease, Head and neck cancer

## Abstract

Head and neck squamous cell carcinoma (HNSCC) still lacks effective targeted treatment. Therefore, exploring novel and robust molecular targets is critical for improving the clinical outcome of HNSCC. Here, we reported that the expression levels of family with sequence similarity 64, member A (FAM64A) were significantly higher in HNSCC tissues and cell lines. In addition, FAM64A overexpression was found to be strongly associated with an unfavorable prognosis of HNSCC. Both in vitro and in vivo evidence showed that FAM64A depletion suppressed the malignant activities of HNSCC cells, and vice versa. Moreover, we found that the FAM64A level was progressively increased from normal to dysplastic to cancerous tissues in a carcinogenic 4-nitroquinoline-1-oxide mouse model. Mechanistically, a physical interaction was found between FAM64A and forkhead box protein M1 (FOXM1) in HNSCC cells. FAM64A promoted HNSCC tumorigenesis not only by enhancing the transcriptional activity of FOXM1, but also, more importantly, by modulating FOXM1 expression via the autoregulation loop. Furthermore, a positive correlation between FAM64A and FOXM1 was found in multiple independent cohorts. Taken together, our findings reveal a previously unknown mechanism behind the activation of FOXM1 in HNSCC, and FAM64A might be a promising molecular therapeutic target for treating HNSCC.

## Introduction

Head and neck cancer (HNC), originating from the mucosal epithelium of the oral cavity, pharynx, and larynx, is the sixth most common cancer worldwide.^1–4^ The most common histological type of HNC is squamous cell carcinoma (HNSCC), which accounts for more than 90% of all HNC cases. The HNSCC burden varies substantially across different countries and regions.^5,6^ Genetic susceptibility, exposure to tobacco carcinogens, excessive alcohol consumption, and human papillomavirus (HPV) infection are well-established risk factors for HNSCC.^7^ Unfortunately, although the head and neck regions are accessible for direct examination, most HNSCC patients are diagnosed at late stages. More importantly, despite the enormous progress that has been achieved in the diagnosis and treatment of HNSCC in the recent decades, the 5-year overall survival rate of HNSCC patients has stagnated at 50%.^8–10^ Therefore, there is an urgent need to identify novel and reliable molecular markers for the clinical management of HNSCC.

Family with sequence similarity 64, member A (FAM64A) was originally identified as a clathrin assembly lymphoid myeloid leukemia gene (CALM)-interacting protein.^11^ FAM64A is tightly associated with cell proliferation, as it was found to be highly expressed in rapidly proliferating cells, but not in non-proliferating cells.^12^ In addition to cell proliferation, FAM64A is actively involved in regulating T17 differentiation as well as inflammation associated cancer and autoimmune disorders.^13^ Abnormal expression of FAM64A has been reported in several types of cancer such as prostate cancer, breast cancer, and pancreatic cancer.^14–16^ However, the potential role of FAM64A in HNSCC is unknown. In addition, understanding of the detailed molecular mechanism accounting for the tumor promoting role of FAM64A is still in its infancy.

In the present study, our results showed that FAM64A was increased in HNSCC tissues and cell lines. FAM64A overexpression was significantly associated with an unfavorable clinical outcome in HNSCC. In addition, the expression level of FAM64A was found to be progressively increased during HNSCC carcinogenesis, indicating its aberrant expression might play a crucial role in the initiation and development of HNSCC. Moreover, knockdown of FAM64A suppressed the malignant phenotypes of HNSCC cells both in vitro and in vivo, and vice versa. Mechanistically, we not only demonstrated that FAM64A directly interacted with FOXM1, and enhanced its transcriptional activity but also, more importantly, provided compelling evidence showing that FAM64A promoted HNSCC tumorigenesis through modulating FOXM1 expression via the FOXM1 autoregulation loop.

## Results

### The clinical significance of FAM64A in HNSCC

**Fig. 1:**
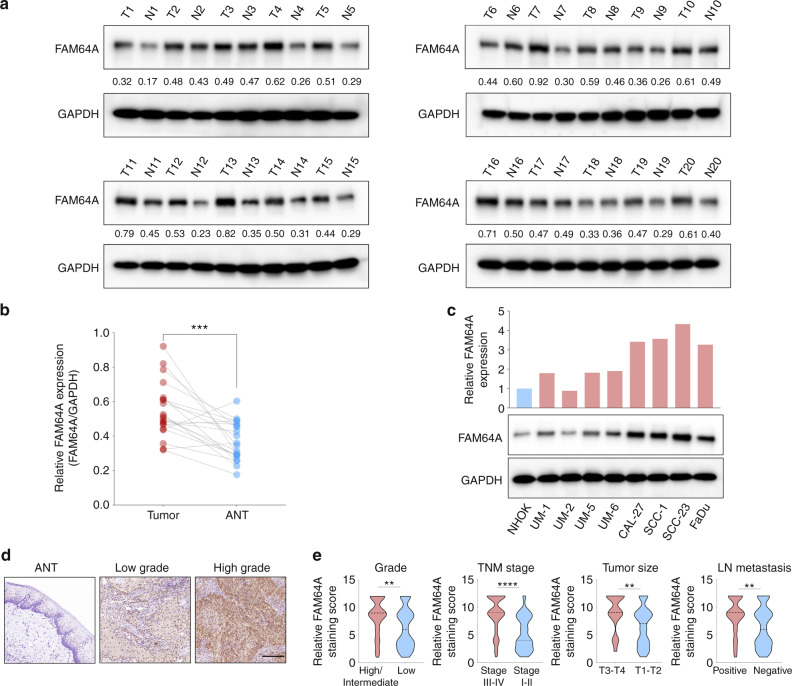
Clinical significance of FAM64A in HNSCC. **a**, **b** Western blot analysis of FAM64A protein expression in 20 HNSCC tumor tissues and paired ANTs. **c** Western blot analysis of FAM64A protein expression in HNSCC cell lines and NHOK. **d**, **e** IHC analysis of FAM64A staining intensity in HNSCC tissues and nontumor tissues. Data are presented as the mean ± SD. **P < 0.01, ***P < 0.001, ****P < 0.0001. Scale bar: 200 μm for (**d**)

To determine differences in FAM64A expression between HNSCC tissues and dysplasia/normal tissues, we first analyzed transcriptome data from TCGA (The Cancer Genome Atlas) and GEO (Gene Expression Omnibus) datasets. Our results showed that FAM64A was consistently overexpressed in HNSCC tissues compared to paired adjacent normal tissues (ANTs) (TCGA HNSCC, GSE127165, and GSE37991) (Fig. S1a–c) or normal control tissues (GSE55550, GSE23036, and GSE30784) (Fig. S1d–f). In addition, in the GSE30784 and GSE85195 datasets, the expression level of FAM64A was markedly higher in tumor tissues than in precancerous lesions (Fig. S1f, g). Higher FAM64A expression was observed in HNSCC patients at stage III–IV or with grade G3–G4 compared to those at stage I–II or with grade G1–G2 (Fig. S1h, i). For the TCGA HNSCC cohort, overall survival (OS) and disease-free survival (DFS) were both significantly worse among patients in the high FAM64A expression group than among those in the low FAM64A expression group (Fig. S1j, k). Similarly, in GSE41643, HNSCC patients with higher FAM64A expression suffered more unfavorable OS (Fig. S1l). We then compared the expression level of FAM64A between HNSCC tumor tissues (*n* = 20) and paired ANTs at the protein level. Our results showed that FAM64A was remarkably higher in tumor tissues than in ANTs (Fig. 1a, b). Moreover, FAM64A was overexpressed in most of the examined HNSCC cell lines (except UM2) compared to the normal cell line NHOK (Fig. 1c). As shown in Fig. 1d, the FAM64A staining intensity was significantly stronger in HNSCC tissues than in normal tissues. More importantly, the staining score of FAM64A was particularly higher in HNSCC patients with lymph node involvement, advanced clinical stage, advanced T stage or poorly differentiated carcinoma (Fig. 1e). We also analyzed the potential clinical significance of FAM64A in other types of cancer in TCGA datasets. FAM64A was overexpressed in breast cancer (BRCA), colon adenocarcinoma (COAD), kidney renal clear cell carcinoma (KIRC), kidney renal papillary cell carcinoma (KIRP), liver hepatocellular carcinoma (LIHC), lung adenocarcinoma (LUAD), and uterine corpus endometrial carcinoma (UCEC) compared to the corresponding ANTs. In addition, FAM64A overexpression was significantly associated with worse OS in these cancer types (Fig. S2a–g).

### Functional role of FAM64A in HNSCC in vitro

**Fig. 2:**
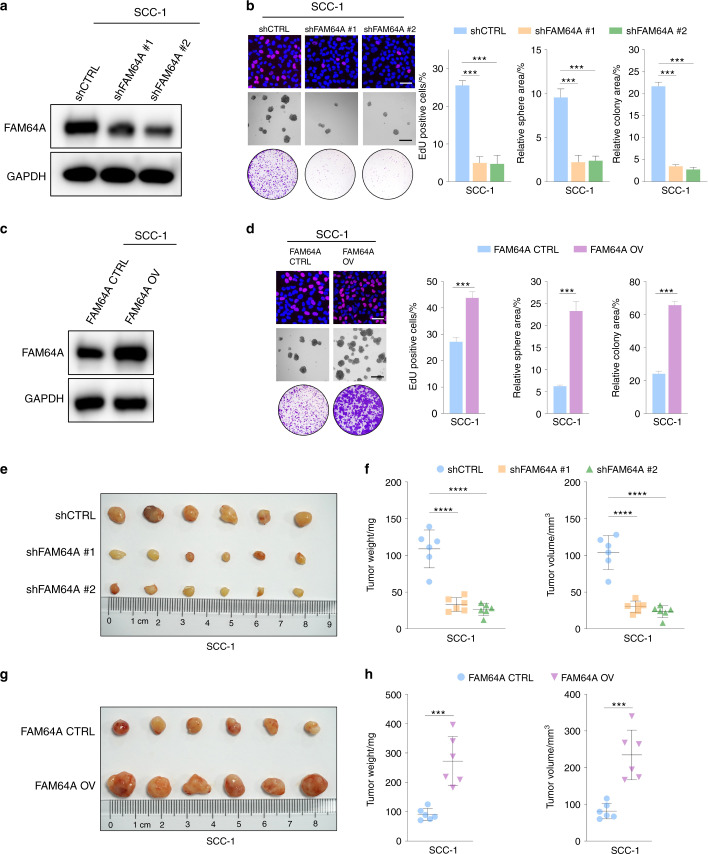
FAM64A promotes the malignant behaviors of HNSCC cells in vitro and in vivo. **a** Validation via western blotting of the depletion effect of shFAM64A in SCC-1 cells. **b** FAM64A depletion significantly reduced the percentage of EdU-positive cells and suppressed the tumor sphere formation capability and colony formation capacity of SCC-1 cells. **c**, **d** The proliferation, tumor sphere formation, and colony formation capacities were significantly enhanced in FAM64A overexpressing cells compared to control cells. **e**, **f** In vivo xenograft studies demonstrated that FAM64A depletion significantly attenuated the size, weight, and volume of tumors derived from SCC-1 cells. **g**, **h** Ectopic expression of FAM64A dramatically increased the size, weight, and volume of tumors derived from SCC-1 cells. Data are presented as the mean ± SD. ***P < 0.001, ****P < 0.0001. Scale bar: 50 μm for upper (**b**) and (**d**) and 200 μm for lower (**b**) and (**d**)

Stable FAM64A knockdown cell lines were generated to study the function of FAM64A in HNSCC (Fig. 2a and Fig. S3a). EdU assay results showed that the percentage of EdU positive HNSCC cells was significantly lower in the FAM64A-knockdown group than in the control group. Tumor sphere formation and colony formation assays demonstrated that FAM64A suppression significantly reduced the size and number of tumor spheres and colonies formed by HNSCC cells (Fig. 2b and Fig. S3b). Since the proliferative and tumor stemness phenotypes were significantly affected by FAM64A inhibition, we next detected markers reflecting proliferation and cancer stemness, and found that the levels of PCNA, CD44, SOX2, and BMI-1 were all markedly decreased upon reduction of FAM64A (Fig. S3c). As shown in Fig. S4a–d, the inhibitory effects of FAM64A downregulation on the malignant behaviors of HNSCC cells were rescued by a shRNA-resistant FAM64A construct. Similarly, the expression levels of proliferation and stemness markers abrogated by shFAM64A were restored by the shRNA-resistant form of FAM64A (Fig. S4e).

Stable and inducible expression of FAM64A was established in HNSCC cells using a tetracycline-inducible expression system. The expression level of FAM64A was increased by tetracycline treatment in a dose-dependent manner (Fig. S5a). As confirmed with the EdU, tumor sphere and colony formation assays, tetracycline enhanced the proliferation, tumor sphere formation and colony formation capabilities of HNSCC cells in a dose-dependent fashion (Fig. S5b–d). As shown in Fig. S5e, western blotting demonstrated dose-dependent induction of PCNA, CD44, SOX2, and BMI-1 with tetracycline in HNSCC cell lines. As expected, compared to the control group, the HNSCC cells in the FAM64A overexpression group had noticeably enhanced malignant phenotypes and higher expression levels of proliferation and stemness markers (Fig. 2c, d and Fig. S6a–c).

### Functional role of FAM64A in HNSCC in vivo

To determine the role of FAM64A in tumor growth in vivo, SCC-1 cells with or without FAM64A depletion were subcutaneously injected into nude mice. The size, volume, and weight of xenograft tumors were significantly lower in the FAM64A knockdown group than in the control group (Fig. 2e, f). In addition, IHC staining demonstrated that downregulation of FAM64A decreased the expression of Ki-67, CD44, and SOX2 in tumor tissues (Fig. S7a, b). Similar findings were observed in SCC-23 cells (Fig. S7c–f). Likewise, nude mice were inoculated with SCC-1 or SCC-23 cells with or without FAM64A overexpression by subcutaneous injection. The size, volume, and weight and the staining intensities of Ki-67, CD44, and SOX2 were significantly higher in the tumors derived from FAM64A-overexpressing HNSCC cells than in tumors derived from control cells (Fig. 2g, h and Fig. S8a–f). A carcinogenic 4-nitroquinoline-1-oxide (4-NQO) model was established to further evaluate the role of FAM64A in the HNSCC tumorigenesis. Our results demonstrated that the level of FAM64A was progressively increased from normal to dysplastic to cancerous tissues, indicating that FAM64A acts as a key player in driving HNSCC onset and progression (Fig. S9a–c).

### FAM64A activates FOXM1 expression via the FOXM1 autoregulation loop

**Fig. 3:**
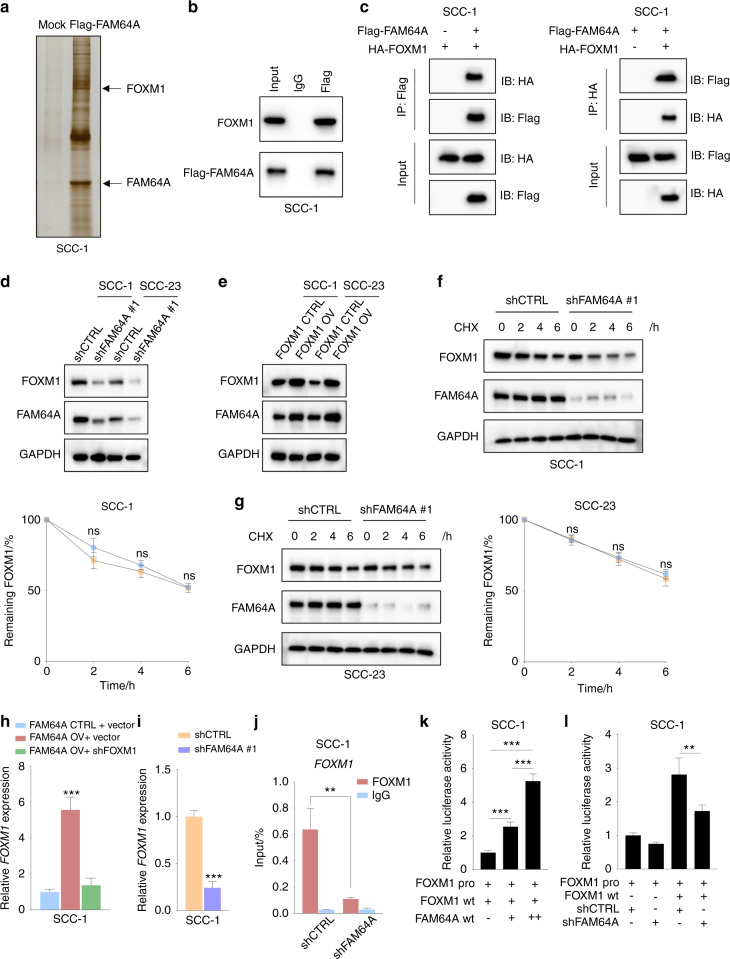
FAM64A interacts with FOXM1, and regulates FOXM1 expression via the FOXM1 autoregulation loop. **a** FAM64A-binding proteins were revealed by immunoblotting and silver staining. **b**, **c** Co-IP assays were performed to further validate the interaction between FAM64A and FOXM1 in HNSCC cells. **d**, **e** FAM64A depletion reduced FOXM1 protein levels in both HNSCC cell lines, and ectopic expression of FAM64A increased FOXM1 levels in SCC-1 and SCC-23 cells. **f**, **g** SCC-1 cells/SCC-23 cells with or without FAM64A depletion were treated with CHX for different periods of time, and the expression level of FOXM1 was detected by immunoblotting. **h** FAM64A upregulation enhanced the expression level of FOXM1 mRNA in SCC-1 cells, and FOXM1 downregulation abrogated these effects. **i** FAM64A depletion reduced the FOXM1 mRNA level in HNSCC cells. **j** CHIP-qPCR results demonstrated that FAM64A depletion significantly reduced the occupancy of FOXM1 on the promoter of FOXM1 in HNSCC cells. **k** FAM64A enhanced FOXM1 transcriptional activity on the FOXM1 promoter in a dose-dependent fashion. **l** FAM64A depletion decreased FOXM1 transcriptional activity on the FOXM1 promoter in HNSCC cells. Data are presented as the mean ± SD. **P < 0.01, ***P < 0.001, ns=not significant

It was reported that FAM64A positively regulates STAT3 activity,^13^ and this regulatory effect was not obvious in HNSCC cells based on our preliminary findings (data not shown). Therefore, Co-IP coupled with MS analysis was performed to identify the proteins potentially associated with FAM64A. The binding proteins of FAM64A were visualized by immunoblotting and silver staining. Interestingly, we found that FOXM1 was one of the potential FAM64A-interacting proteins (Fig. 3a). Immunoprecipitation of FAM64A pulled down FOXM1, and reciprocal Co-IP also demonstrated an interaction between FAM64A and FOXM1 (Fig. 3b, c). To explore whether the interaction between FAM64A and FOXM1 was functional, we first investigated the effects of FAM64A alteration on the expression level of FOXM1. Our results showed that knockdown of FAM64A significantly decreased the expression level of FOXM1 and that FAM64A overexpression remarkably enhanced the FOXM1 protein level (Fig. 3d, e). In addition, western blotting results showed that the expression level of FOXM1 protein was lower in the tumor tissues formed by FAM64A-knockdown cells, and the opposite results were observed when FAM64A was overexpressed (Fig. S10a, b). Moreover, IHC staining demonstrated that the staining intensity of FOXM1 in xenograft tumor tissues was significantly lower in the FAM64A depletion group, and dramatically higher in the FAM64A overexpression group (Fig. S10c–f). Furthermore, we explored the correlation between FAM64A and FOXM1 using publicly assessable databases. The results showed compelling evidence that FAM64A was positively correlated with FOXM1 in TCGA HNSCC cohort and multiple GEO datasets including GSE127165, GSE30784, GSE37991, GSE40774, GSE41613, GSE65858, and GSE117973 (Fig. S11). These findings suggested that FAM64A interacts with FOXM1 and regulates the expression level of FOXM1. To further elucidate the underlying molecular mechanism, we first hypothesized that FAM64A might increase the FOXM1 level by enhancing its stability. After treatment with cycloheximide (CHX), the expression level of FOXM1 changed little in FAM64A depleted cells compared to control cells, indicating that FAM64A alteration did not affect the degradation of FOXM1 (Fig. 3f, g). We then investigated the effects of FAM64A upregulation or downregulation on the expression of FOXM1 mRNA. Interestingly, the FOXM1 mRNA level was markedly increased by FAM64A overexpression, and the enhancing effects were diminished by the suppression of FOXM1 (Fig. 3h). In addition, the expression of FOXM1 mRNA was dramatically decreased in shFAM64A-treated cells (Fig. 3i). CHIP-qPCR showed that FAM64A suppression markedly decreased the occupancy of FOXM1 to the promoter of FOXM1 in SCC-1 cells (Fig. 3j). To determine the contribution of FAM64A on FOXM1 transcriptional activity, HNSCC cells were co-transfected with FOXM1 and increasing amounts of FAM64A expression vectors, and the results showed that FAM64A increased FOXM1 transcriptional activity on the FOXM1 promoter in a dose-dependent manner (Fig. 3k). In addition, FAM64A depletion reduced FOXM1 transcriptional activity on the FOXM1 promoter (Fig. 3l). These findings demonstrated that FAM64A promotes the expression of FOXM1 through the FOXM1 autoregulation loop.

### FAM64A regulates FOXM1 transcriptional activity

**Fig. 4:**
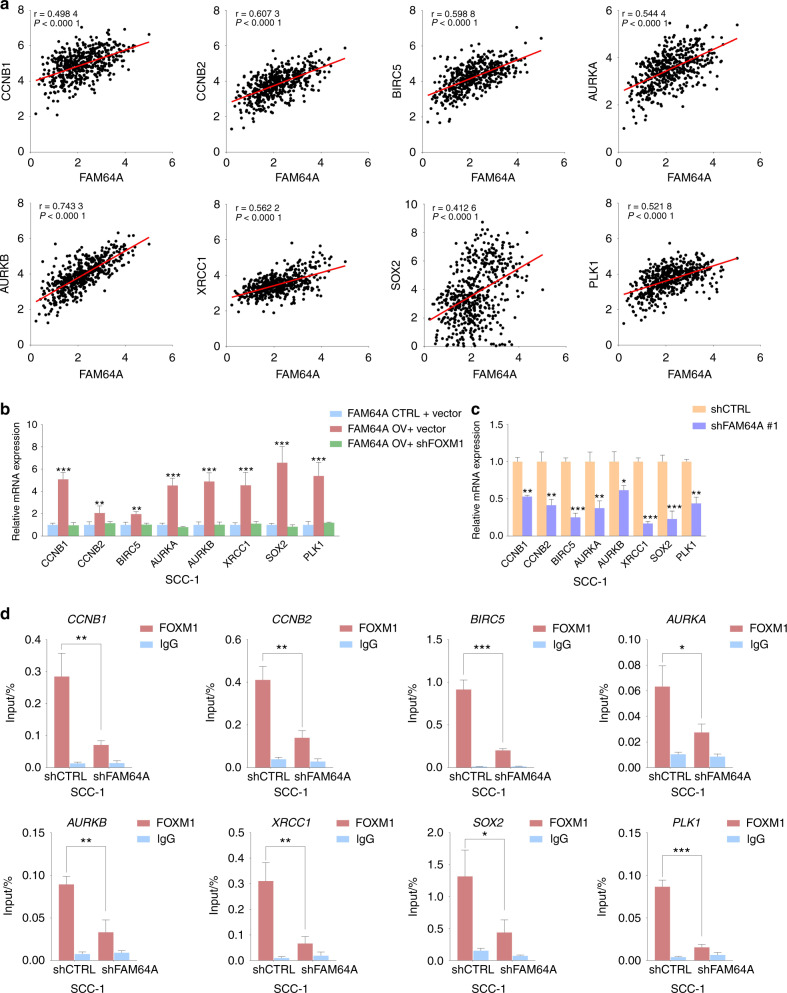
FAM64A regulates FOXM1 transcriptional activity in HNSCC cells. **a** FAM64A was found to be positively correlated with FOXM1 target genes including *CCNB1*, *CCNB2*, *BIRC5*, *AURKA*, *AURKB*, *XRCC1*, *SOX2*, and *PLK1* in TCGA HNSCC cohort. **b** qRT-PCR results revealed that FAM64A overexpression enhanced the expression levels of FOXM1 target genes in SCC-1 cells, and FOXM1 depletion abrogated these promotive effects. **c** FAM64A depletion suppressed the expression levels of FOXM1 target genes in SCC-1 cells. **d** CHIP-qPCR demonstrated that FAM64A downregulation markedly reduced the occupancy of FOXM1 on the promoters of *CCNB1*, *CCNB2*, *BIRC5*, *AURKA*, *AURKB*, *XRCC1*, *SOX2*, and *PLK1* in SCC-1 cells. Data are presented as the mean ± SD. *P < 0.05, **P < 0.01, ***P < 0.001

We first evaluated the correlation between FAM64A and FOXM1 targets using the TCGA HNSCC cohort and GSE127165. Our results showed that a positive correlation between FAM64A and representative FOXM1 targets including *CCNB1*, *CCNB2*, *BIRC5*, *AURKA*, *AURKB*, *XRCC1*, *SOX2*, and *PLK1* was consistently observed in both independent cohorts (Fig. 4a and Fig. S12a). The expression levels of these FOXM1 targets were significantly upregulated by overexpression of FAM64A, and the promotive effects were abrogated by downregulation of FOXM1 (Fig. 4b and Fig. S12b). In addition, the levels of FOXM1 targets were markedly reduced in FAM64A depleted HNSCC cells (Fig. 4c and Fig. S12c). Then CHIP-qPCR was used to determine whether FAM64A regulated the expression levels of FOXM1 targets by modulating FOXM1 transcriptional activity. The results showed that FAM64A depletion significantly reduced the occupancy of FOXM1 on the promoters of *CCNB1*, *CCNB2*, *BIRC5*, *AURKA*, *AURKB*, *XRCC1*, *SOX2*, and *PLK1* in SCC-1 cells (Fig. 4d).

### FOXM1 overexpression rescues the oncostatic effects of FAM64A depletion

**Fig. 5:**
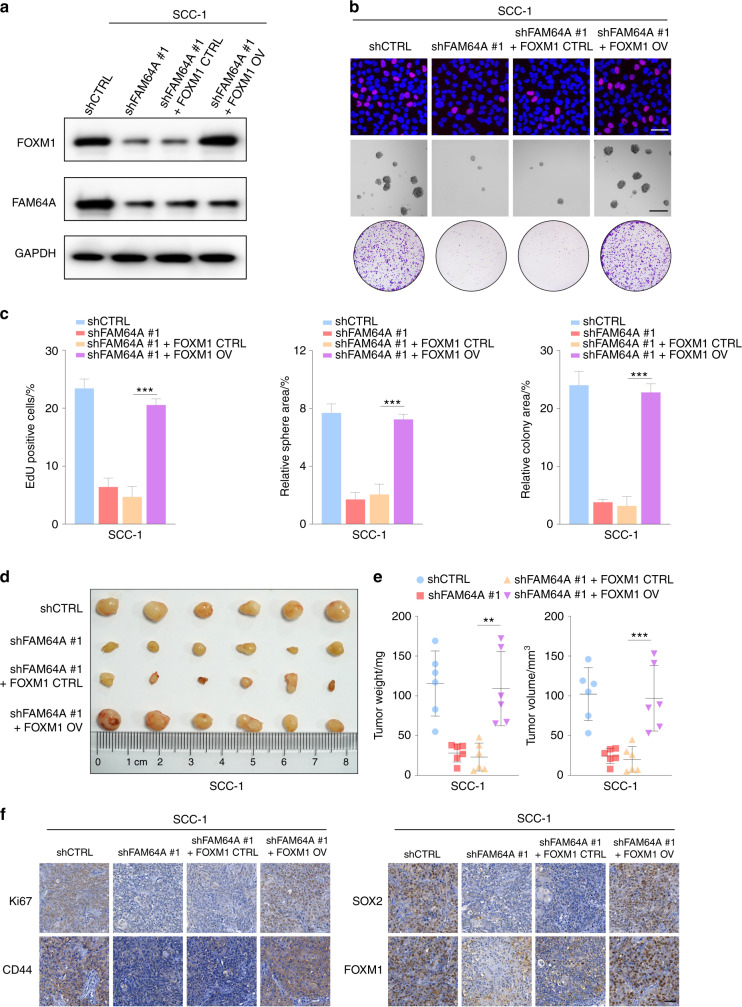
FOXM1 overexpression rescued the tumor suppressive effects of FAM64A depletion in vitro and in vivo. **a** Western blot analysis of FOXM1 and FAM64A protein expression in SCC-1 cells with the indicated modifications. **b**, **c** The proliferation, tumor sphere formation and colony formation capacities of SCC-1 cells were suppressed by FAM64A depletion. These suppressive effects were reversed by ectopic expression of FOXM1. **d**, **e** FAM64A depletion repressed the growth of tumors derived from SCC-1 cells, and the inhibitory effects were rescued by FOXM1 overexpression. **f** The Ki-67, CD44, SOX2, and FOXM1 staining intensity were decreased in xenograft tumor tissues formed by shFAM64A-treated SCC-1 cells, and FOXM1 overexpression abrogated these effects. Data are presented as the mean ± SD. **P < 0.01, ***P < 0.001. Scale bar: 50 μm for upper (**b**) and (**f**), 200 μm for lower (**b**)

Since we demonstrated that FAM64A can regulate FOXM1 expression through the FOXM1 autoregulation loop and modulate the transcriptional activity of FOXM1, we further investigated whether FOXM1 overexpression reversed the inhibitory effects of FAM64A depletion. The in vitro findings revealed that FOXM1 upregulation rescued the proliferative capacity of HNSCC cells with FAM64A reduction. Likewise, the clonogenic and tumor-initiating potential of shFAM64A-treated cells was restored by overexpression of FOXM1 (Fig. 5a–c and Fig. S13a–c). The expression levels of PCNA, CD44, SOX2, and BMI-1 inhibited by FAM64A suppression were also rescued by ectopic expression of FOXM1 (Fig. S13d). The in vivo results showed that FAM64A depletion significantly reduced tumor size, volume and weight, and that FOXM1 overexpression restored these suppressive effects. In addition, IHC staining demonstrated that the FAM64A depletion-induced downregulation of Ki-67, SOX2, CD44 and FOXM1 was abrogated by FOXM1 overexpression (Fig. 5d–f and Fig. S14a–e).

### FAM64A expression is positively associated with FOXM1 levels in HNSCC tissues and the prognostic value of the FAM64A-FOXM1 axis in HNSCC

**Fig. 6:**
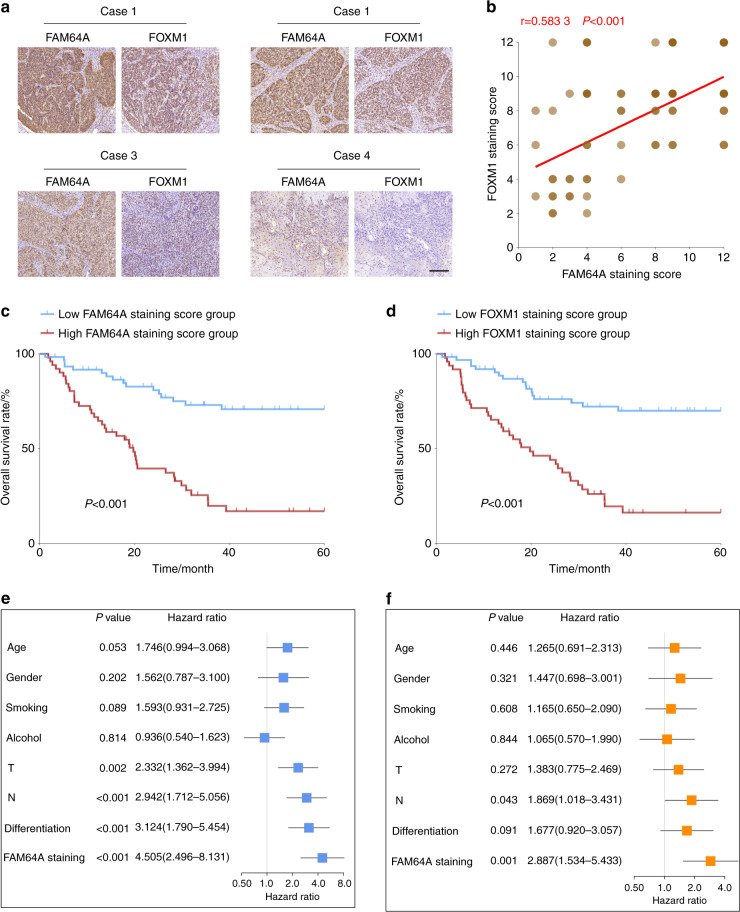
FAM64A expression is positively correlated with FOXM1 expression in HNSCC tumor tissues and the prognostic value of the FAM64A-FOXM1 axis in HNSCC. **a**, **b** The expression level of FAM64A in HNSCC tumor tissues was positively correlated with FOXM1 expression. **c** HNSCC patients in the high FAM64A IHC staining score group suffered worse OS than those in the low FAM64A IHC staining score group. **d** Patients in the high FOXM1 IHC staining score group had more unfavorable OS than those in the low FOXM1 IHC staining score group. **e**, **f** Univariate analysis revealed that FAM64A staining score was strongly associated with HNSCC patient OS, and the multivariate analysis demonstrated that it was an independent prognostic factor for HNSCC. Scale bar: 200 μm for (**a**)

To further validate the clinical association between FAM64A and FOXM1, we analyzed the expression patterns of FAM64A and FOXM1 in HNSCC specimens. Not surprisingly, consistent with the results observed in the TCGA HNSCC cohort and multiple GEO datasets, a positive correlation between FAM64A protein expression and FOXM1 protein expression was found by IHC staining of HNSCC samples (Fig. 6a, b). We then analyzed the prognostic value of FAM64A and FOXM1 in HNSCC. Survival analysis revealed that patients with higher FAM64A IHC scores suffered worse OS than those with lower FAM64A IHC scores (Fig. 6c). Similarly, HNSCC patients with higher FOXM1 staining intensity also had more unfavorable OS (Fig. 6d). The prognostic value of FAM64A in HNSCC was further evaluated by univariate and multivariate analyses. Univariate analysis showed that the FAM64A staining score was significantly correlated with the OS of HNSCC patients, and multivariate analysis demonstrated that it was an independent prognostic factor for HNSCC (Fig. 6e, f).

## Discussion

**Fig. 7:**
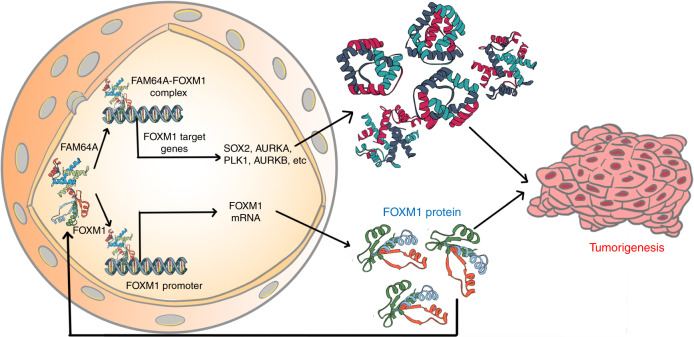
A graphical model for FAM64A-mediated tumorigenesis in HNSCC. FAM64A promotes HNSCC tumorigenesis by increasing FOXM1 transcriptional activity and activating FOXM1 expression via the FOXM1 autoregulation loop.

HNSCC remains a major public health problem worldwide. The clinical outcome is unfavorable for patients with HNSCC, especially for those with recurrent and/or metastatic HNSCC.^17–19^ Unfortunately, the current treatment strategies have achieved only modest improvement in HNSCC survival.^20,21^ Therefore, it is critical to develop novel targeted therapies. In this study, we identified that FAM64A is overexpressed in HNSCC, and that upregulation of FAM64A is associated with unfavorable clinical outcomes in HNSCC. In addition, FAM64A is progressively increased during HNSCC carcinogenesis. More importantly, as illustrated in Fig. 7, we provided compelling evidence showing that FAM64A promotes HNSCC tumorigenesis by increasing FOXM1 transcriptional activity and activating FOXM1 expression via the FOXM1 autoregulation loop.

To the best of our knowledge, we are the first to report the prognostic value of FAM64A in HNSCC. Our analyses of TCGA datasets also suggested that FAM64A was overexpressed and associated with an unfavorable survival time in KIRC, KIRP, LIHC and UCEC, which has not been reported to date. These findings indicate that the tumor- promoting role of FAM64A might be present across different cancer types. In the 4-NQO induced HNSCC model, the expression level of FAM64A progressively increased during HNSCC carcinogenesis. These findings were consistent with the results showing that FAM64A is overexpressed in precancerous lesions compared to normal tissues. Therefore, FAM64A might actively modulate HNSCC tumorigenesis at a very early stage.

Regarding the functional role of FAM64A in HNSCC, we provided strong evidence that FAM64A acts as a tumor promoter in HNSCC both in vitro and in vivo. FAM64A not only enhanced the proliferative capacity of HNSCC cells but also promoted HNSCC stemness. Although FAM64A is closely correlated with malignant behaviors in cancer cells, the detailed molecular mechanisms are poorly understood. Similarly, downregulation of FAM64A suppressed the malignant activities of breast cancer cells, and the opposite findings were observed when FAM64A was overexpressed.^22^ Ectopic expression of FAM64A enhanced the proliferation, migration and invasion capabilities of osteosarcoma cells, and the tumor promoting effects of FAM64A were mediated by miR-493.^23^ FAM64A downregulation was also reported to suppress the proliferation of lung cancer cells.^24^ Although FAM64A is closely correlated with the malignant behaviors of cancer cells, the detailed molecular mechanisms are poorly understood.

Mechanistically, we demonstrated that FAM64A interacted with FOXM1, which subsequently enhanced FOXM1 transcriptional activity and activated the expression level of FOXM1 via an autoregulation loop. FOXM1 is a member of the evolutionarily conserved forkhead box transcription factor family, which has been shown to play numerous regulatory roles in eukaryotes.^25–28^ Abnormal expression of FOXM1 has been reported in a number of human malignancies including HNSCC.^29–32^ Increased FOXM1 levels were found at the early stage of HNSCC, and FOXM1 played an important role in inducing genomic instability and epigenetic changes.^33,34^ Not surprisingly, multiple independent studies have presented strong evidence that FOXM1 functions as a tumor promoter in HNSCC tumorigenesis,^35–38^ which is in consistent with our findings that FOXM1 is upregulated in HNSCC tissues and that FOXM1 overexpression is associated with worse survival in HNSCC. Therefore, FOXM1 is a master regulator that is involved in HNSCC tumorigenesis. Our studies showed that FAM64A is a functional upstream regulator of FOXM1, which further established the critical role of FAM64A in the initiation and development of HNSCC. Previously, FAM64A was reported to facilitate binding of STAT3 to the promoters of its target genes and consequently increased STAT3 transcriptional activity.^13^ FAM64A is mainly located in the nucleus, and might acts as a molecular chaperone to functionally activate transcription factors, thereby modulating many important biological processes.

Our analyses of the TCGA HNSCC cohort and multiple GEO datasets revealed a positive correlation between FAM64A and FOXM1, as well as between FAM64A and FOXM1-target genes in HNSCC tumor tissues. More importantly, the clinical significance of our findings was evidenced by the positive association of FAM64A overexpression with FOXM1 upregulation in HNSCC specimens. These findings reveal a previously unknown mechanism by which FOXM1 and its regulated pathway are activated in HNSCC in a FAM64A-dependent manner. The FAM64A-FOXM1 axis not only plays a crucial role in the progression of HNSCC, but also might serve as a novel and reliable prognostic molecular signature contributing to individualized treatment and improving the unfavorable prognosis of HNSCC.

FOXM1 is an oncogene and maintains cancer features by transactivating genes with tumor promotion potential. In addition, overexpression of FOXM1 has been closely associated with inherent or acquired drug resistance in multiple cancer types, which is a major contributor to treatment failure.^32,39^ Therefore, pharmacological inhibition of FOXM1 is an effective therapeutic strategy for cancer. However, targeting transcriptional factor is particularly challenging. In addition, due to their off-target actions, the effects of currently available FOXM1 inhibitors are still not desirable.^39^ In this study, our results demonstrate that FAM64A physically interacts with FOXM1, and regulates FOXM1 activity via the autoregulation loop. Developing efficient and selective agents against FAM64A might provide a good alternative for suppressing FOXM1 activity. It should be pointed out that further studies are warranted to determine the functional activities of FAM64A and FOXM1 using HNSCC patient-derived cells, which are very important to reflect the close interactions between FAM64A and FOXM1 in the tumor microenvironment.

Collectively, our study demonstrates that FAM64A is overexpressed in HNSCC specimens and that high FAM64A expression is significantly correlated with unfavorable prognosis in HNSCC patients. In addition, FAM64A is actively involved in HNSCC carcinogenesis. Moreover, FAM64A promotes the malignant behaviors of HNSCC cells both in vitro and in vivo. Furthermore, FAM64A interacts with FOXM1, which leads to increased FOXM1 transcriptional activity and promotes FOXM1 expression through the FOXM1 autoregulation loop. The discovery of the crucial control of FOXM1 by FAM64A might provide promising approaches for the clinical intervention in HNSCC.

## Materials and methods

### Patients and clinical samples

The study protocol regarding the reutilization of clinical samples and medical information was approved by the Ethics Committee of the First Affiliated Hospital of Fujian Medical University (FMU [2015]084-1). Written Informed consent was obtained from all patients. All procedures were undertaken in accordance with Declaration of Helsinki. The HNSCC tissue specimens, adjacent normal tissues and the corresponding clinical information were obtained from the First Affiliated Hospital of Fujian Medical University. The detailed clinicopathological features of HNSCC cohort were listed in Supplementary Table S1.

### Cell lines and cell culture

UMSCC-1 (SCC-1), UTSCC-23 (SCC-23), UMSCC-5, UMSCC-6, UM1, UM2, CAL-27, and FaDu cell lines were maintained in the Dulbecco’s modified eagle medium (DMEM) supplemented with 10% fetal bovine serum, 100 U·mL^−1^ penicillin, and 100 μg·mL^−1^ streptomycin. Normal human oral keratinocytes (NHOKs) were cultured in EpiLife media supplemented with the human keratinocyte growth supplement (Invitrogen, Carlsbad, CA, USA). All cells were cultured at 37 °C in a humidified incubator with 5% CO_2_.

### EdU (5-ethynyl-2’-deoxyuridine) assay

The Click-iT™ EdU Cell Proliferation Kit for Imaging (Invitrogen) was used to detect cell proliferation. In brief, the cells were incubated in 10 µmol·L^−1^ EdU labeling solution for 2 h. After fixation with 4% paraformaldehyde and permeabilization with 0.5%Triton X-100, Click-iT® Plus reaction cocktail was added to each well and the samples were incubated for 30 min in the dark. After washing in PBS, Hoechst 33342 solution was used to stain the cell nucleus. Images were captured with an inverted fluorescence microscope (Leica Microsystems, Wetzlar, Germany).

### Colony formation assay

Cells were seeded a six-well plate at 3000 cells per well density. After 14 days of incubation, the cells were fixed with 4% paraformaldehyde and stained with 0.5% crystal violet.

### Sphere formation assay

The sphere formation assay was performed as previously described. Briefly, cells were grown in ultra-low attachments plates and cultured in DMEM/F12 supplemented with 1% B27 supplement (Invitrogen), 1% N_2_ supplement (Invitrogen), 20 ng·mL^−1^ EGF and 10 ng·mL^−1^ bFGF. The formed spheres were taken with an inverted microscope (Leica Microsystems).

### Production of lentiviral particles and cell transduction

The oligonucleotide sequences targeting FAM64A were inserted into LV3-pGLV-h1-GFP-puro vector (GenePharma, Shanghai, China). The shRNA sequences are listed in Supplementary Table S2. The full-length of human FAM64A gene and FOXM1 gene were cloned to pGCL-GFP vectors to construct the FAM64A overexpression vector and FOXM1 overexpression vector, respectively. For the tetracycline-inducible lentiviral expression, FAM64A gene was cloned into the inducible Tet-on lentiviral vector. Then the cells were treated with different doses of tetracycline. HEK-293T cells were transfected with recombinant lentiviral vectors, packaging plasmids and envelope plasmids to produce lentiviruses. The cancer cells were infected with lentiviruses at a multiplicity of infection of 30.

### Quantitative reverse transcription polymerase chain reaction (qRT-PCR)

Total RNA was extracted from cells using the Quick-RNA™ kit (Zymo Research Corp, Irvine, CA, USA) according to the manufacturer’s protocols. The reverse transcription was performed using SuperScript™III First-Strand Synthesis SuperMix (Invitrogen). The qRT-PCR was carried out on a CFX96 Real-Time PCR detection system (Bio-Rad, Hercules, CA, USA) using Light Cycler 480@ SYBR Green I MasterMix (Roche, Applied Science, Indianapolis, IN, USA). The relative quantification of genes was determined with 2^−ΔΔCt^ method using GAPDH as an internal control. The qRT-PCR primer sequences were listed in Supplementary Table S2.

### Western blotting

Tissues and cells were lysed in RIPA buffer (Beyotime, Shanghai, China) in the presence of Protease Inhibitor Cocktail. The protein concentration was determined with a BCA Protein Assay Kit (Beyotime). Equal amounts of protein samples (20–40 μg) were loaded and separated on a 4%–20% SDS-PAGE gels (Invitrogen) and transferred onto a PVDF membrane using a Trans-Blot Turbo system (Bio-Rad). After blocked with blocking buffer (EpiZyme, Shanghai, China) for 30 min at room temperature, the PVDF membranes were incubated with primary antibodies at 4 °C overnight. After washing five times in TBST solution for 5 min each time, the membranes were hybridized with the HRP-linked secondary antibodies (Proteintech, Chicago, IL, USA) at room temperature for 1 h. The membranes were then washed with TBST (5 min, five times) and the intensity of the bands was detected with Amersham ECL Prime Western Blotting Detection Reagent (Cytiva, Marlborough, MA, USA). GAPDH was used as an internal control for western blot analyses. The primary antibodies for SOX2 (dilution 1:800), BMI-1(dilution 1:1000), CD44 (dilution 1:2000), PCNA (dilution 1:4000) and GAPDH (dilution 1:5000) were from Proteintech. The FAM64A primary antibody (dilution 1:1000) was from Novus Biologicals (Littleton, CO, USA). The FOXM1 primary antibody (dilution 1:1000) was from Cell Signaling Technology (Danvers, MA, USA).

### Luciferase reporter assay

The FOXM1 promoter reporter vector was co-transfected with FOXM1 wild type (wt) plasmid, FAM64A wt plasmid, shFAM64A and shCTRL alone or in combination into HNSCC cells using the Lipofectamine 3000 (Invitrogen) transfection reagent according to the manufacturer’s instructions. After 24 h of incubation, the relative luciferase activities were determined using a Dual-Luciferase Assay System (Promega, Madison, WI, USA) following the manufacturer’s protocol.

### Chromatin immunoprecipitation (ChIP)-qPCR

The ChIP assays were performed with a ChIP assay kit (Millipore, Billerica, MA, USA) as previously described.^40^ The cells were cross-linked with 1% formaldehyde for 10 min at room temperature to cross-link proteins to DNA, and the reaction was terminated by adding 125 mmol·L^−1^ glycine. After washing twice with cold PBS, the cells were lysed in RIPA buffer and sonicated to generate DNA fragments. The cross-linked chromatin was immunoprecipitated with anti-FOXM1 overnight at 4 °C. The CHIP samples were then un-crosslinked and the DNA was purified. The DNA pellets were resuspended in ultra-pure water and subjected to real-time PCR. The primer sequences used for ChIP-qPCR are available on request.

### LC-MS/MS

The immunocomplexes were separated by SDS-PAGE, followed by silver staining. The candidate bands were digested with sequencing-grade trypsin (Promega) and subjected to MS/MS analysis for protein identification.

### Co-immunoprecipitation (Co-IP) assay

The cells with indicated treatment were lysed in RIPA buffer (Beyotime) at 4 °C for IP and western blotting. After centrifugation at 14000 × *g* for 20 min at 4 °C, the supernatants were incubated with primary antibodies to prepare the immunocomplexes with gentle rocking overnight at 4 °C. Then the immunocomplexes were incubated with the pre-washed protein A/G magnetic beads (EpiZyme) at 4 °C for 8 h with gentle rocking. After washing with the buffer solution, the antigen-antibody-bead complexes were resuspended with SDS sample buffer. The samples were heated to 100 °C for 10 min for denaturation and then subjected to western blotting. The anti-IgG, anti-Flag and anti-HA primary antibodies were from Proteintech.

### Immunohistochemistry (IHC)

After being deparaffinized in xylene and rehydrated, the formalin-fixed paraffin-embedded (FFPE) sections were blocked in goat serum for 2 h at room temperature. Then the specimens were probed with primary antibodies at 4 °C overnight. After washing with three changes of PBS, the slides were incubated with HRP-conjugated secondary antibodies for 1 h at room temperature. Subsequent detection of the staining signal was conducted with DAB kit, counterstained with hematoxylin solution, dehydrated and mounted. For the quantitative IHC analysis, the total staining score of targeted proteins equals to the staining intensity (on a scale of 0–3: negative=0, weak=1, moderate=2, and strong=3) × the percentage of cells stained (on a scale of 0–4: 0 = 0%, 1 = 1%–25%, 2 = 26%–50%, 3 = 51%–75%, and 4 = 76%–100%), resulting in a score range of 0–12. Two experienced pathologists who were blind to this study scored and validated all sections. In addition, the IHC staining was performed by the same experienced technician under the same experimental condition. To minimize the impact of intratumor heterogeneity on interpretation of IHC results, at least five sections were stained and scored for each specimen.

### Animal experiments

The animal experiments were approved by the Animal Ethics Committee of Southern Medical University. For the generation of 4-nitroquinoline 1-oxide (4-NQO) induced HNSCC model, six-week-old C57BL/6 mice were treated with 50 μg·mL^−1^ 4-NQO in the drinking water for 16 weeks and then given with normal drinking water for another 8–10 weeks. For the construction of xenograft mouse model, 2 × 10^6^ SCC-1 or SCC-23 cells with indicated modifications were injected to subcutaneously injected into the dorsal flank of 6-week-old BALB/c nude mice. The mice were bred under specific pathogen-free conditions. After 4 weeks, mice were sacrificed and tumors were harvested. The tumor size and weight were measured and recorded. The collected tumor tissues were processed for IHC analysis.

### Exploring the clinical significance of FAM64A in cancer with public databases

The datasets comparing the gene expression profiles between HNSCC tissues and normal tissues/precancerous lesions were retrieved from NCBI GEO database (https://www.ncbi.nlm.nih.gov/geo/). The accession numbers were GSE127165, GSE37991, GSE55550, GSE23036, GSE30784, and GSE85195. Similarly, the microarray data and corresponding clinical data of GSE41613 were downloaded from GEO database. For TCGA data analysis, the transcriptome data and clinical information of all TCGA tumor types were downloaded from The National Cancer Institute Genomic Data Commons (NCI-GDC) (https://gdc.cancer.gov/). The best cutoff point for dividing the cancer patients into high and low FAM64A expression groups were obtained using the X tile software (https://medicine.yale.edu/lab/rimm/research-/software/).^41^

### Statistical analysis

All data were presented as the mean ± standard deviation. The significance of the differences was evaluated through one-way ANOVA or Student’s *t*-test. Kaplan–Meier method was used for survival analysis, and differences in survival were estimated using the log-rank test. The univariate and multivariate analyses were performed to identify prognostic factors associated with overall survival. The correlation between two variables were analyzed with Pearson or Spearman correlation analysis. *P* < 0.05 was considered as statistically significant. The statistical analyses were performed with Graphpad Prism 9.0 (GraphPad Software, San Diego, CA, USA).

## Supplementary information

Supplementary Information

## Data Availability

The datasets generated during and/or analysed during the current study are available from the corresponding author on reasonable request.
