# Supplementary material for: FAM64A promotes HNSCC tumorigenesis by mediating transcriptional autoregulation of FOXM1
Source: Int J Oral Sci. 2022 May 10;14:25. doi: 10.1038/s41368-022-00174-4 (PMC9091245; doi:10.1038/s41368-022-00174-4)
Supplement: Supplementary file 1 — Supplementary Information [file 41368_2022_174_MOESM1_ESM.pdf]

Supplementary Information

FAM64A promotes HNSCC tumorigenesis by mediating transcriptional autoregulation of FOXM1

Xinyuan Zhao[1, #], Huan Chen[1, #], Yu Qiu[2*], Li Cui[1, 3, *]

[1]Stomatological Hospital, Southern Medical University, Guangzhou, China 510280

[2]Department of Oral and Maxillofacial Surgery, the First Affiliated Hospital, Fujian Medical University, Fuzhou, China 350005

[3]School of Dentistry, University of California, Los Angeles, Los Angeles, California, United States 90095

[#]These authors contribute equally to this work.

[*]Correspondence:

Yu Qiu, Department of Oral and Maxillofacial Surgery, the First Affiliated Hospital, Fujian Medical University, Fuzhou, China 350005. Email: qy97@163.com, Tel: 0591-87982098, Fax: 0591-87982098

Li Cui, Stomatological Hospital, Southern Medical University, Guangzhou, China 510280; School of Dentistry, University of California, Los Angeles, Los Angeles, California, United States 90095. Email: licui@smu.edu.cn, Tel: 020-81602494, Fax: 020-81602494

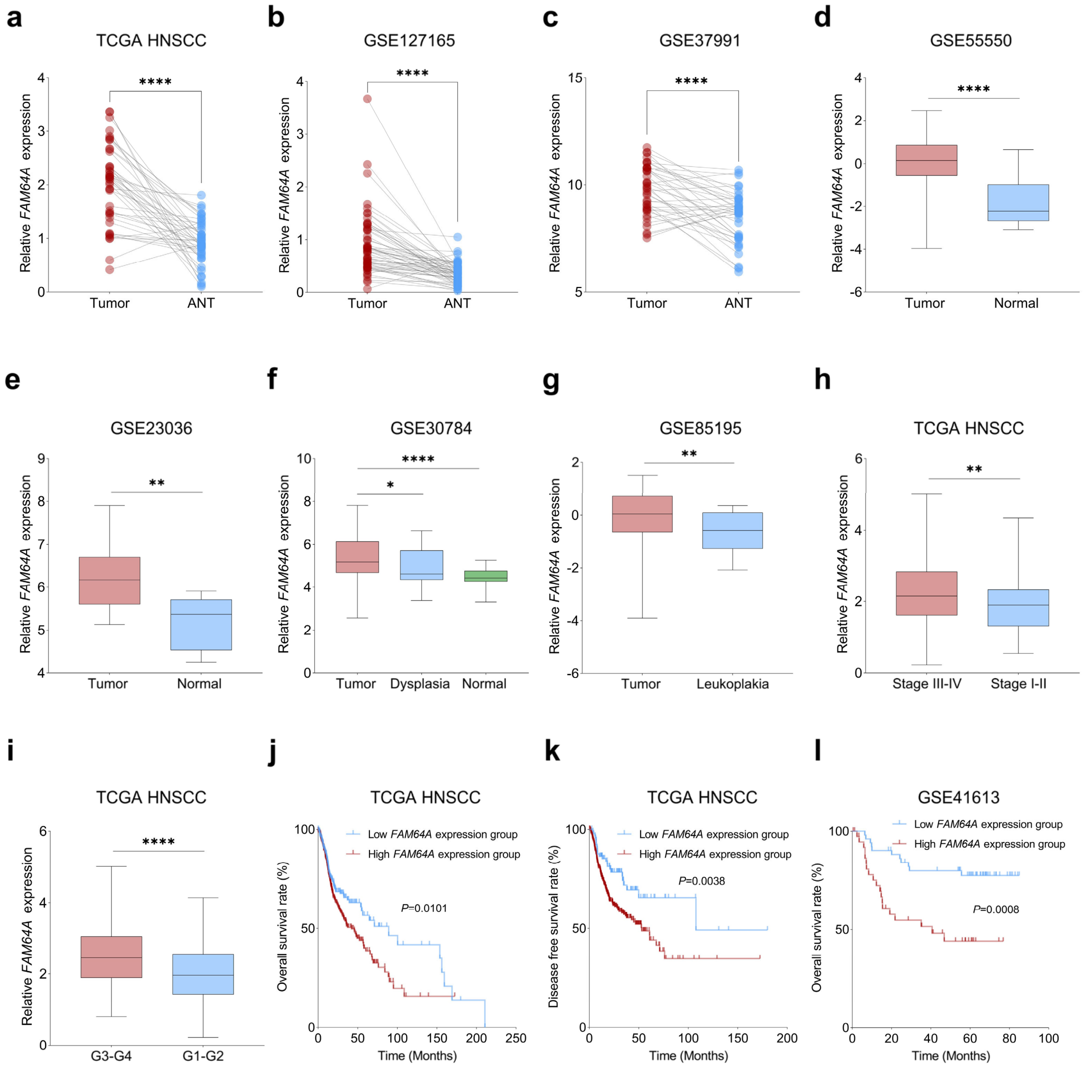

Fig. S1 The clinical significance of FAM64A in HNSCC based on analysis of public datasets. **a-c** The expression level of FAM64A in HNSCC tumor tissues and paired ANTs. **d, e** The expression level of FAM64A in HNSCC tissues and normal control tissues. **f** The expression pattern of FAM64A in tumor tissues, dysplasia tissues and normal control tissues. **g** The level of FAM64A in tumor tissues and precancerous lesions. **h, i** In the TCGA HNSCC cohort, patients at the advanced stages or with grade G3-G4 had higher FAM64A levels than those at the early stages or with grade G1-G2, respectively. **j, k** In the TCGA HNSCC cohort, patients in the high FAM64A expression group suffered worse OS and DFS than those in the low FAM64A expression group. **l** In GSE41613, the HNSCC patients with higher FAM64A expression had shorter OS than those with lower FAM64A expression. Data are presented as the mean ± SD. *$P < 0.05$, **$P < 0.01$, *** $P < 0.001$, ****$P < 0.0001$.

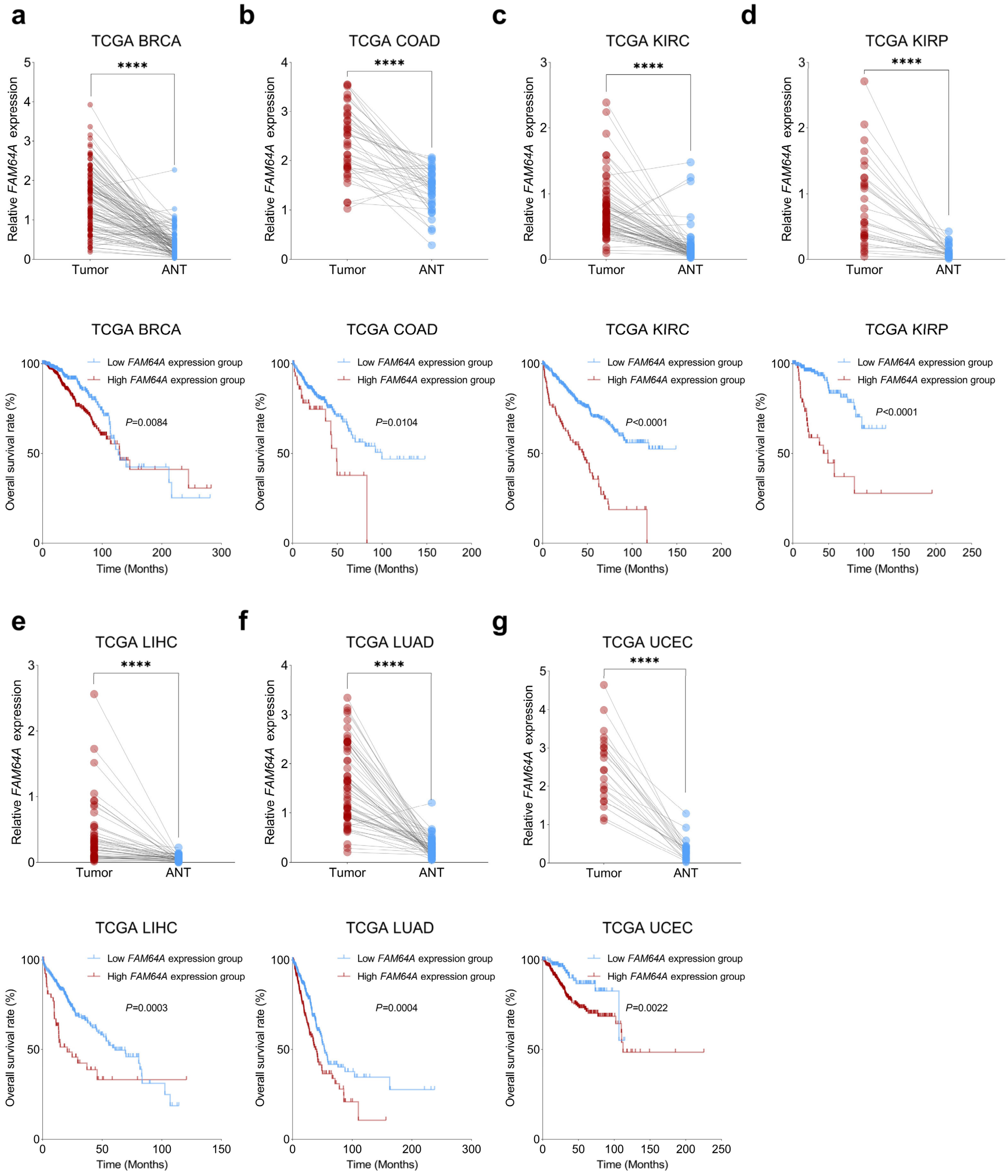

Fig. S2 The clinical significance of FAM64A in human malignancies based on the TCGA datasets. **a-g** Compared to the corresponding ANTs, the expression level of FAM64A was significantly increased in BRCA, COAD, KIRC, KIRP, LIHC, LUAD and UCEC. More importantly, the patients with higher FAM64A expression suffered significantly worse OS than those with lower FAM64A expression in these types of cancer. ****$P < 0.0001$.

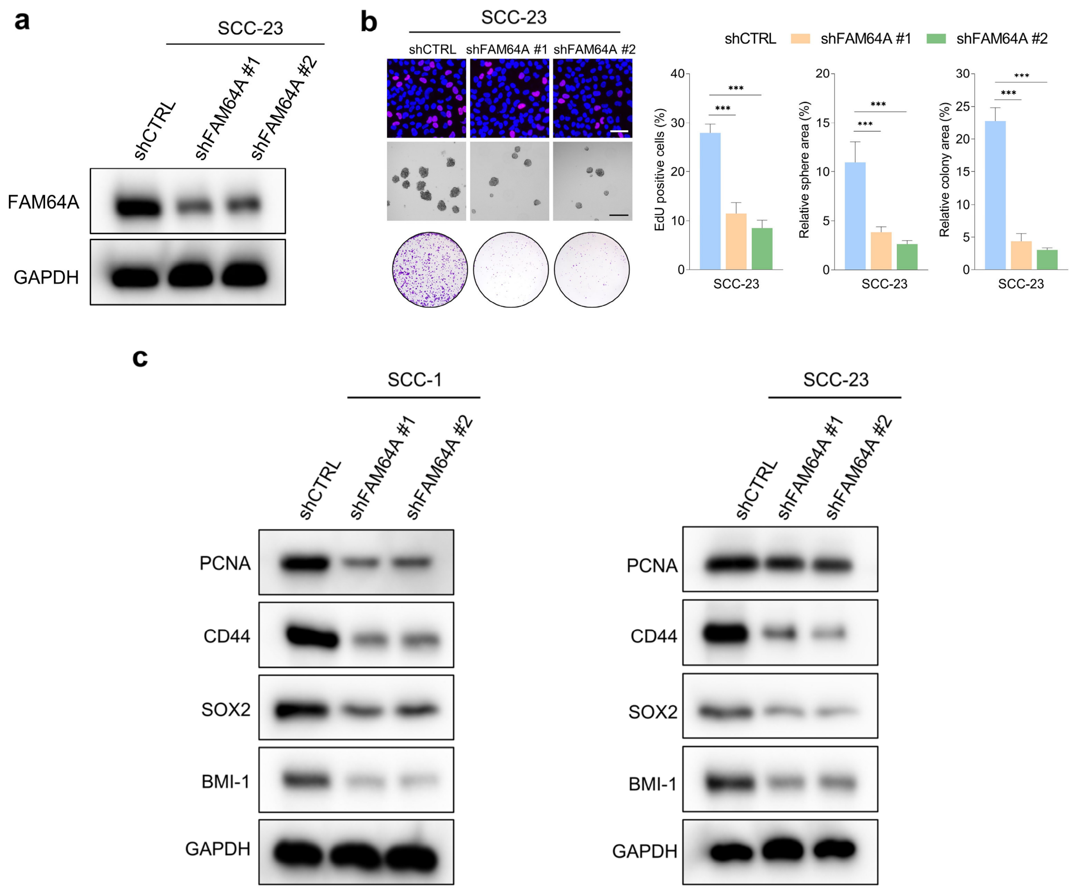

Fig. S3 FAM64A depletion suppressed the malignant behaviors of HNSCC cells *in vitro*. **a** Validation of the depletion effect of shFAM64A by western blotting in SCC-23 cells. **b** FAM64A depletion inhibited the proliferation, tumor sphere formation and colony formation capabilities of SCC-23 cells. **c** Knockdown of FAM64A decreased the levels of PCNA, CD44, SOX2 and BMI-1 in both SCC-1 and SCC-23 cells. Data are presented as the mean ± SD. *** $P < 0.001$. Scale bar: 50 μm for upper **(b)** and 200 μm for lower **(b)**.

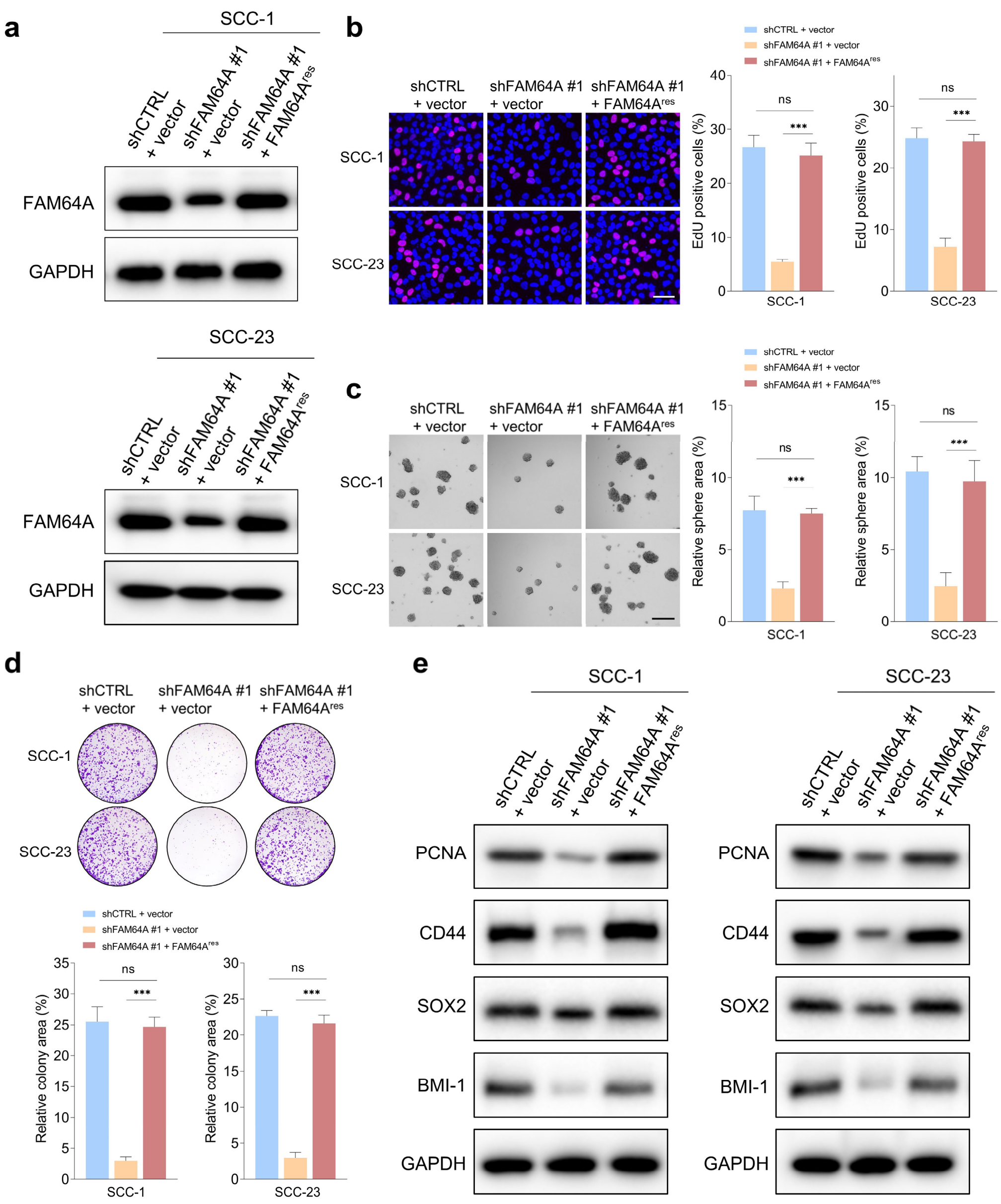

Fig. S4 The tumor suppressive effects elicited by FAM64A depletion were abrogated by reconstituted expression of a shRNA resistant form of FAM64A. **a** Western blot of FAM64A protein expression in HNSCC cells with indicated modifications. **b-d** FAM64A depletion reduced the proliferation, tumor sphere formation and colony formation capacities of HNSCC cells, and these suppressive effects were diminished by reconstituted expression of a shRNA resistant form of FAM64A. (f) The suppressive effects elicited by FAM64A depletion on the expression of proliferation and stemness markers were abrogated by reconstituted expression of a shRNA resistant form of FAM64A. Data are presented as the mean ± SD. ***$P < 0.001$, ns=not significant. Scale bar: 50 μm for **(b)** and 200 μm for **(c)**.

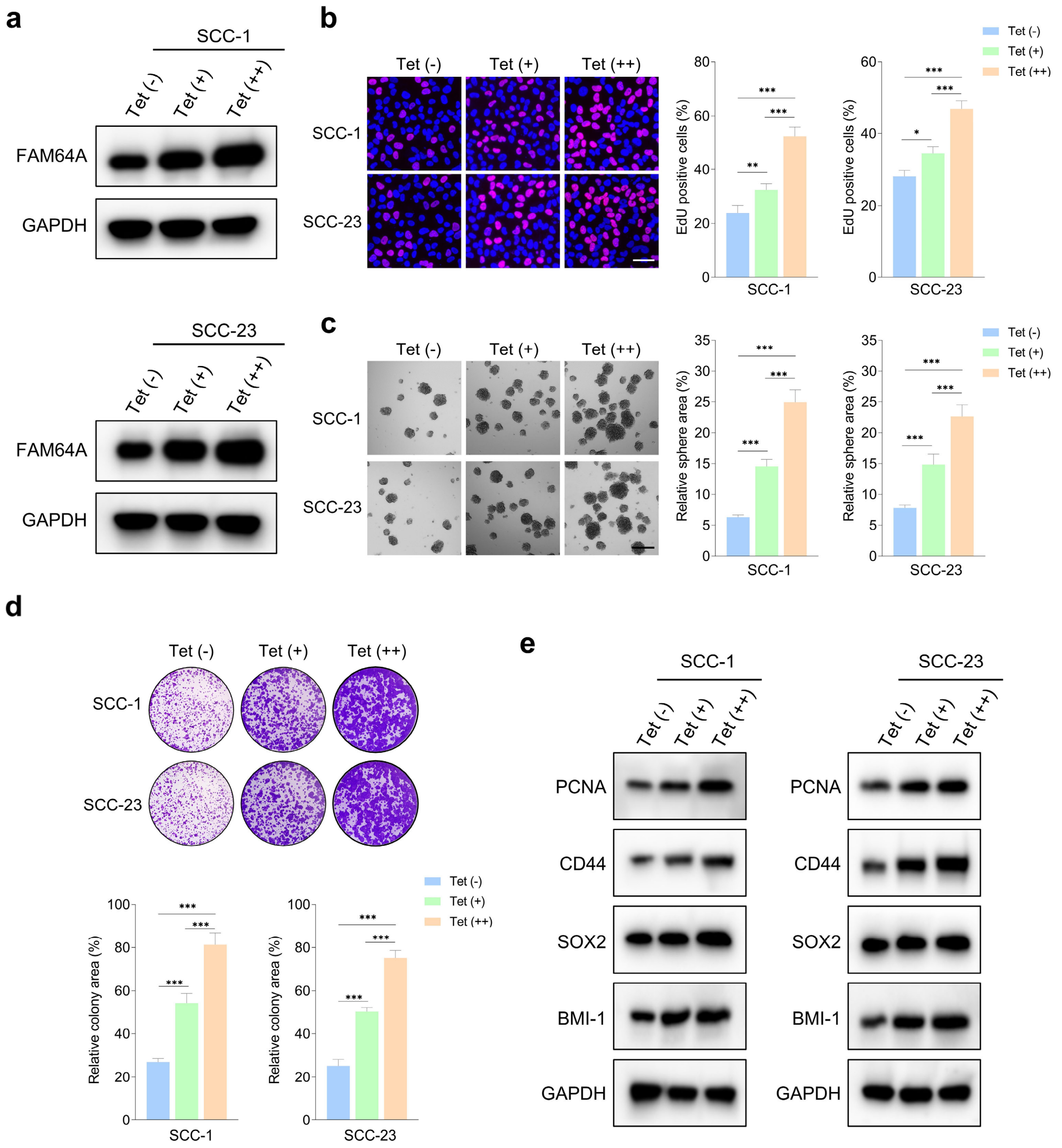

Figure S5 FAM64A overexpression with Tet-on inducible lentiviral vectors promoted the malignant activities of HNSCC cells *in vitro*. **a** Western blot analysis of FAM64A level in HNSCC cells that carry the FAM64A expression-inducible Tet-on lentiviral vector following treatment with low (1 μg/ml) and high (2 μg/ml) dosage of tetracycline. **b-d** Tetracycline treatment promoted the proliferation, tumor sphere formation and colony formation capabilities of HNSCC cells in a dosage-dependent manner. **e** Tetracycline treatment increased the levels of PCNA, CD44, SOX2 and BMI1 in HNSCC cell lines in a dose-dependent manner. Data are presented as the mean ± SD. *$P < 0.05$, **$P < 0.01$, ***$P < 0.001$. Scale bar: 50 μm for **(b)** and 200 μm for **(c)**.

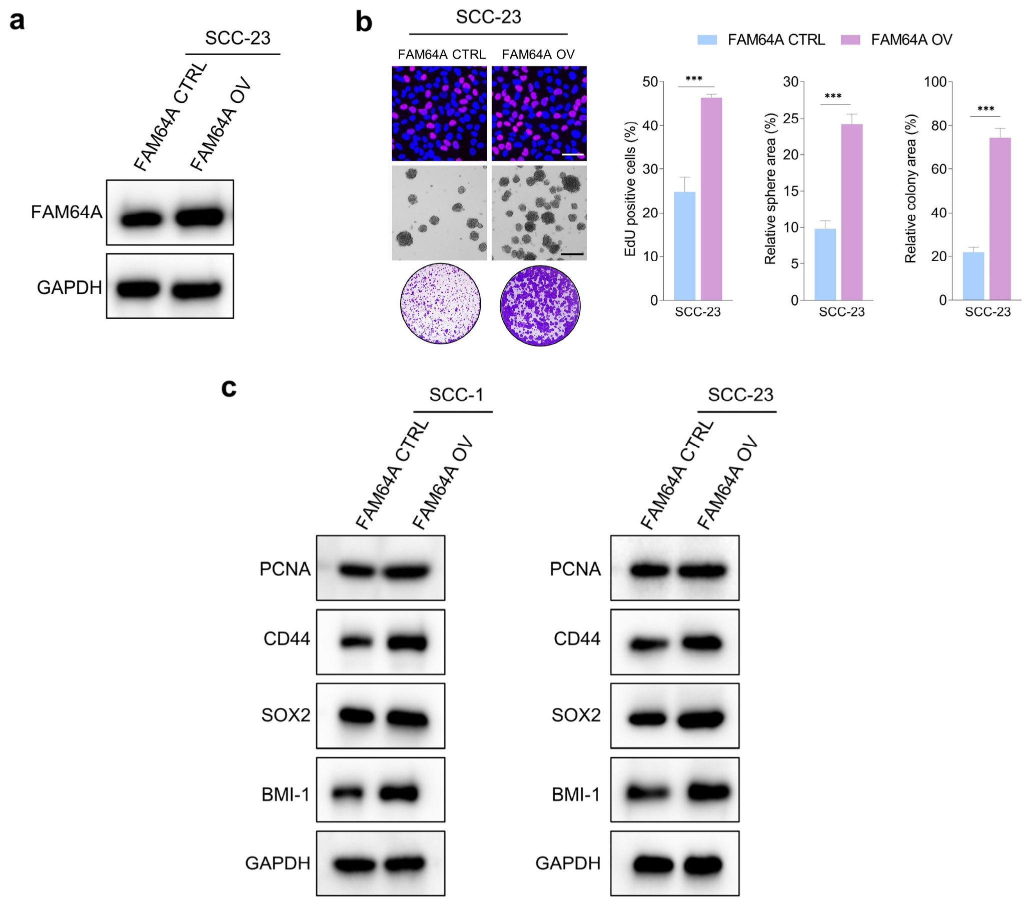

Figure S6 FAM64A overexpression enhanced the malignant activities of HNSCC cells *in vitro*. **a** Western blot analysis of FAM64A level in HNSCC cells with expressing wild type FAM64A. **b** The proliferation, tumor sphere formation and colony formation capacities were significantly enhanced in FAM64A overexpressing cells compared to the control cells. **c** Ectopic expression of FAM64A increased the levels of PCNA, CD44, SOX2 and BMI-1 in SCC-1 and SCC-23 cells. Data are presented as the mean ± SD. ***$P < 0.001$. Scale bar: 50 μm for upper **(b)** and 200 μm for lower **(b)**.

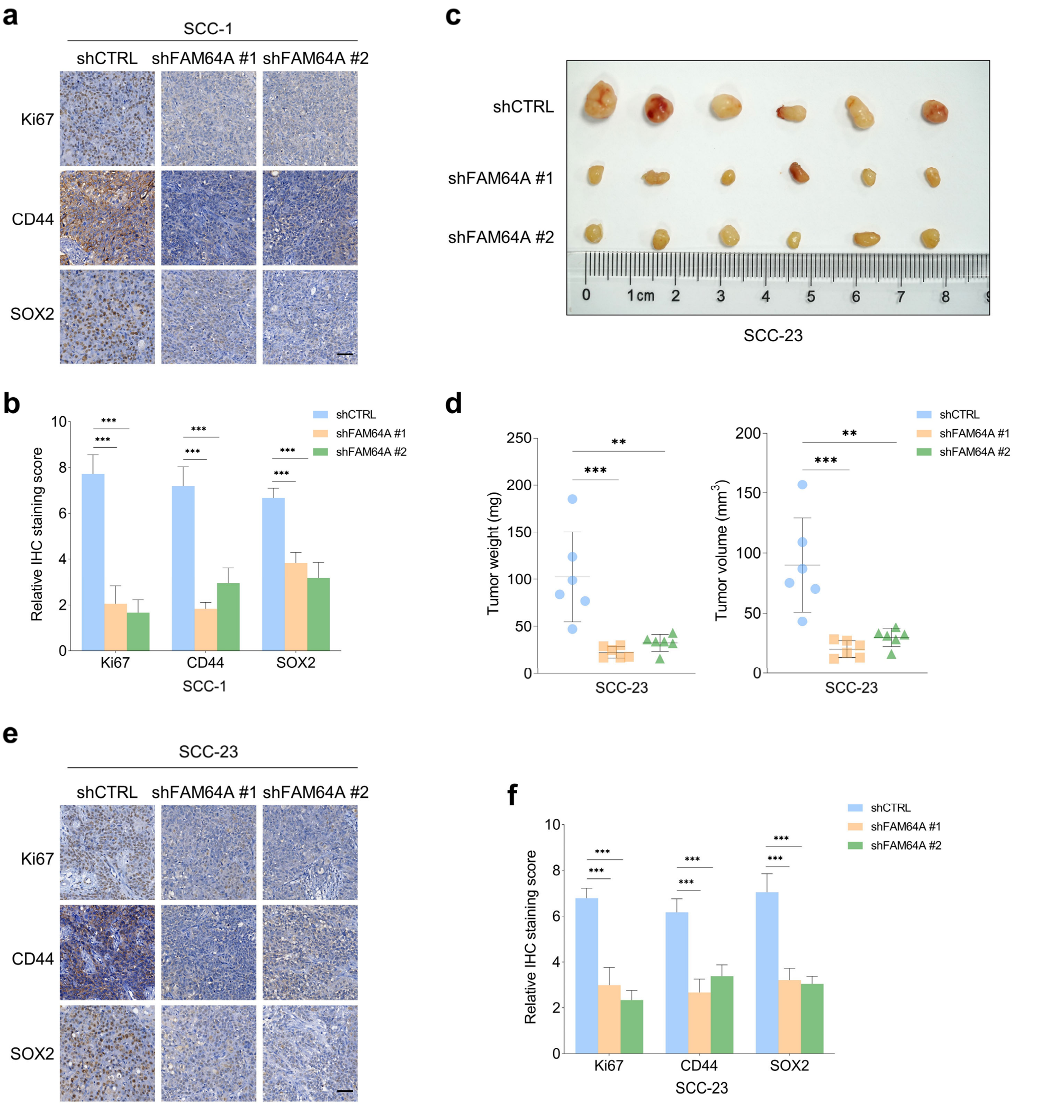

Figure S7 FAM64A depletion suppressed tumor growth *in vivo*. **a, b** Ki-67, CD44 and SOX2 staining intensities were remarkably lower in the xenograft tumor tissues formed by FAM64A-depleted SCC-1 cells. **c-f** In SCC-23 cells, knockdown of FAM64A suppressed the tumor growth and the levels of Ki-67, CD44 and SOX2 in xenograft tumor tissues. Data are presented as the mean ± SD. $^{**}P < 0.01$, $^{***}P < 0.001$. Scale bar: 50 μm for **(a)** and **(e)**.

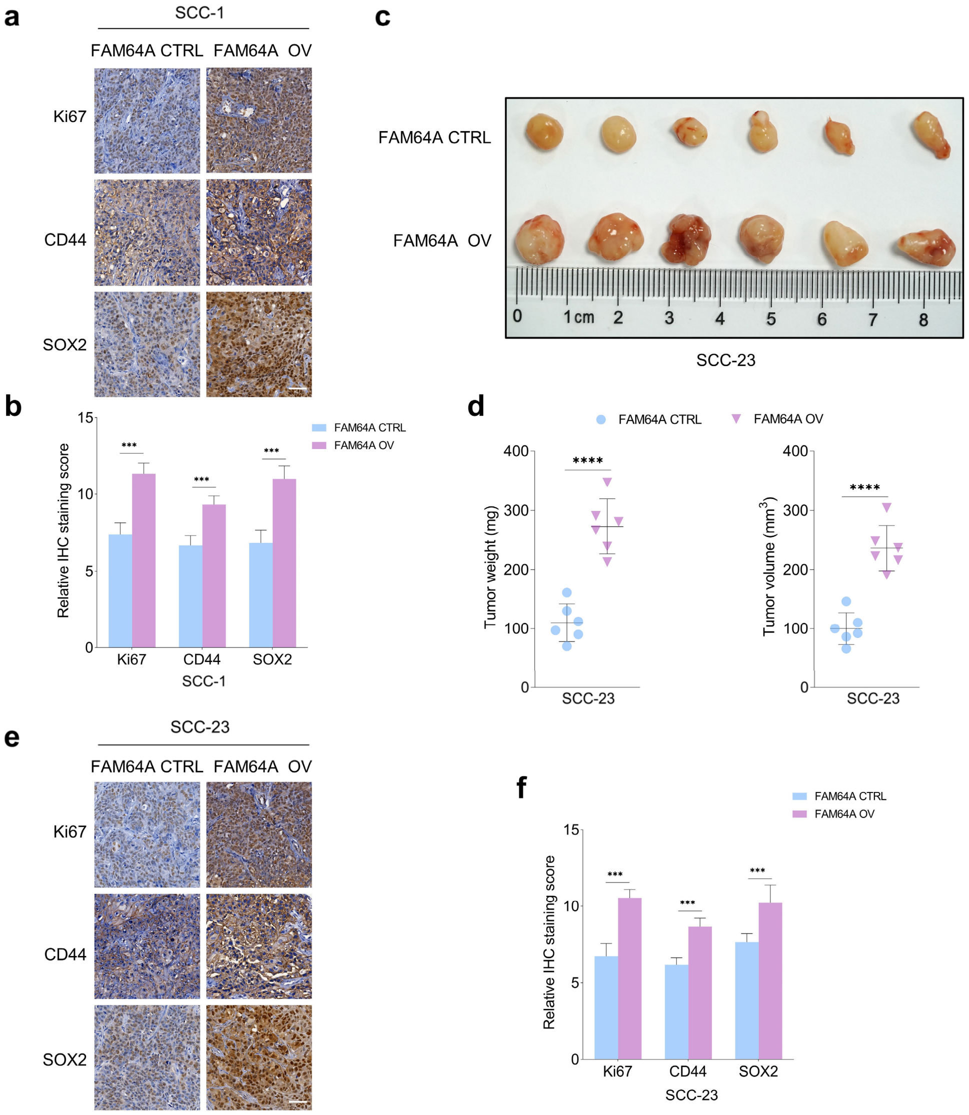

Figure S8 FAM64A overexpression promoted tumor growth *in vivo*. **a, b** The staining intensities of Ki-67, CD44 and SOX2 were significantly higher in the xenograft tumor tissues formed by FAM64A-overexpressing SCC-1 cells. **c-f** In SCC-23 cells, ectopic expression of FAM64A promoted tumor growth in mice model, and enhanced the staining intensities of Ki-67, CD44 and SOX2 in the xenograft tumor tissues. Data are presented as the mean ± SD. ***P < 0.001, ****P < 0.0001. Scale bar: 50 μm for **(a)** and **(e)**.

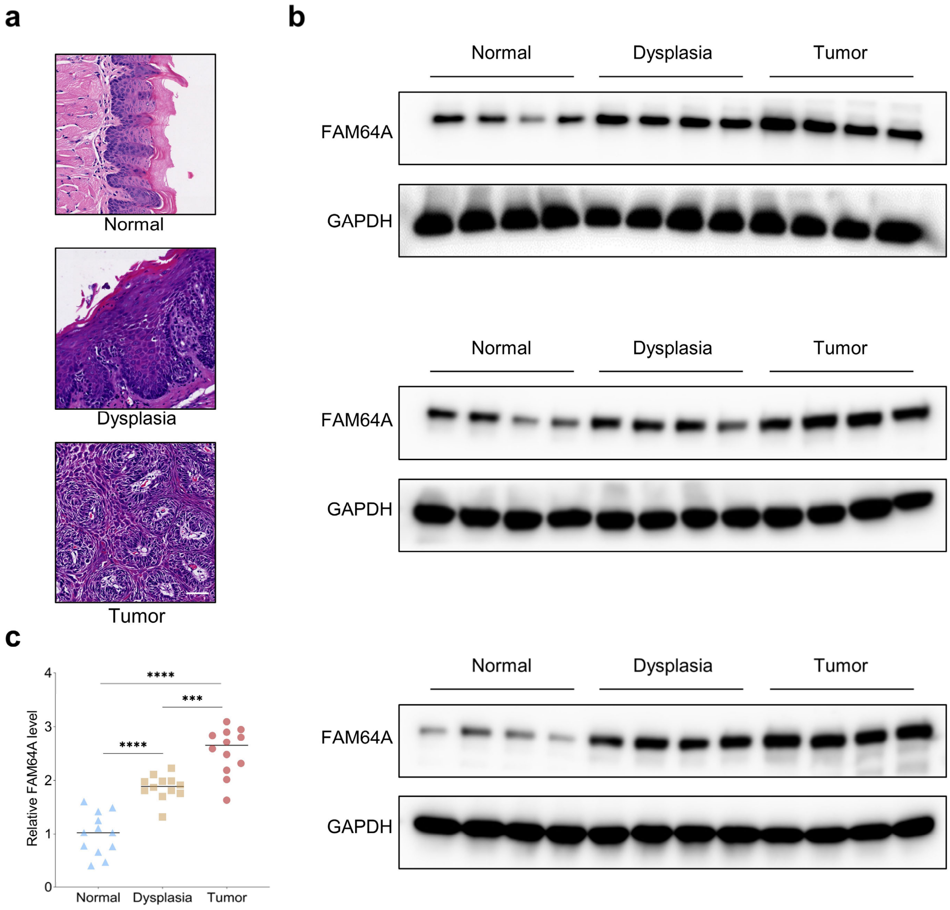

Figure S9 FAM64A was significantly increased in the carcinogenic 4-NQO mouse model. **a** Representative H&E-stained normal tissues, dysplasia tissues and tumor tissues. **b, c** The expression level of FAM64A was progressively increased from normal to dysplastic to cancerous tissues in the carcinogenic 4-NQO mouse model. Data are presented as the mean ± SD. *** $P < 0.001$, ****$P < 0.0001$. Scale bar: 50 μm.

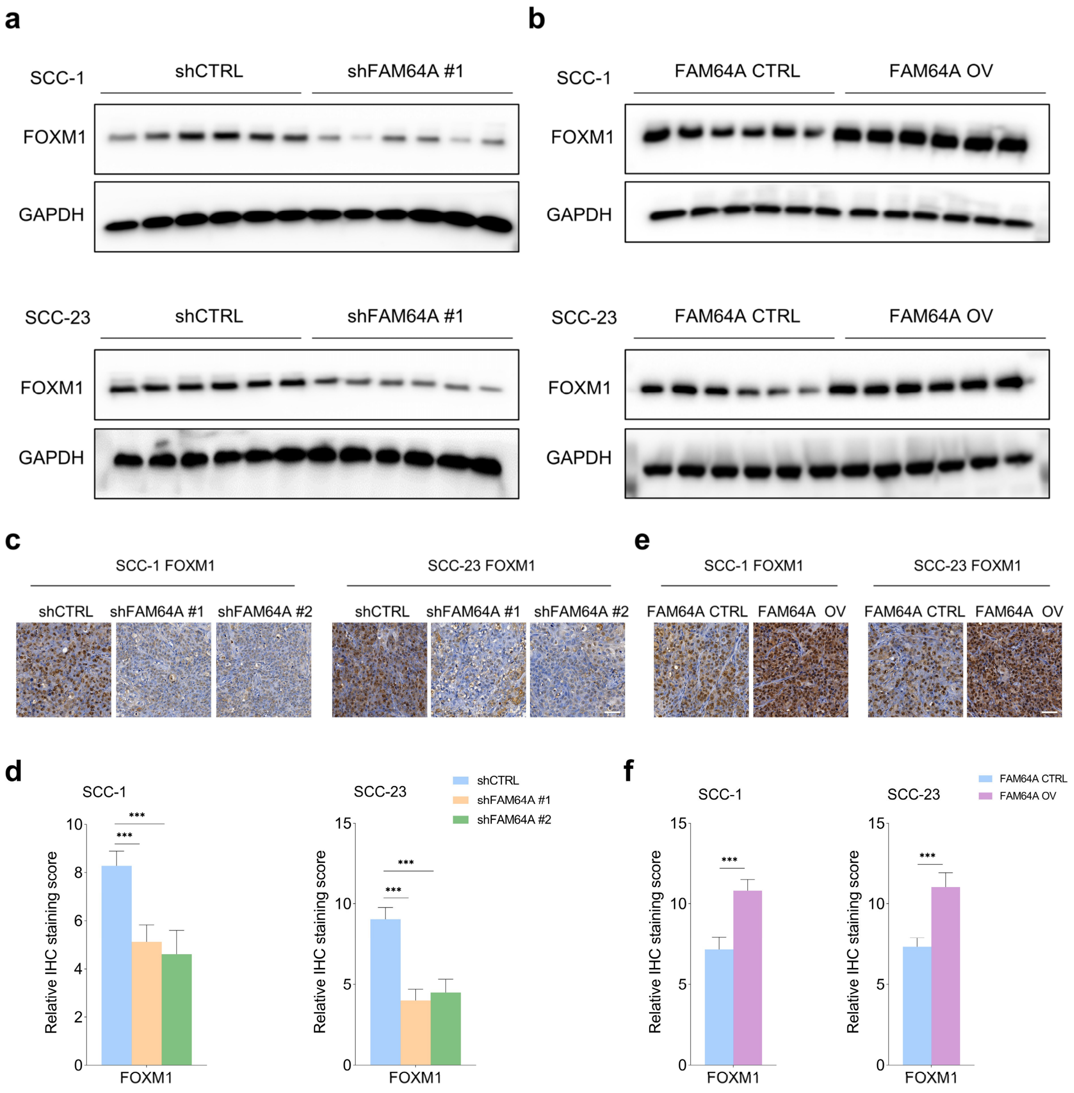

Figure S10 FAM64A regulated FOXM1 expression *in vivo*. **a, b** Western blot assays showed that the protein level of FOXM1 was significantly reduced in xenograft tumor tissues formed by FAM64A depleted SCC-1 and SCC-23 cells, and oppositive results were observed when FAM64A was overexpressed. **c, d** FAM64A depletion reduced FOXM1 staining intensity in xenograft tumor tissues. **e, f** The staining intensity of FOXM1 was markedly higher in xenograft tumor tissues formed by FAM64A-overexpressing HNSCC cells. Data are presented as the mean ± SD. *** $P < 0.001$. Scale bar: 50 μm.

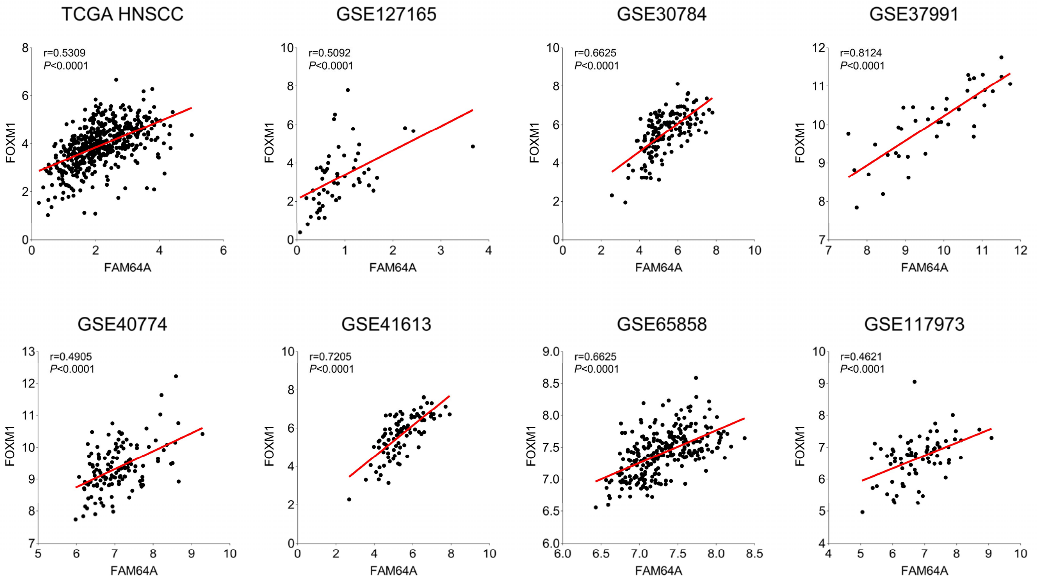

Figure S11 FAM64A positively correlated with FOXM1 expression in multiple HNSCC cohorts. FAM64A was positively correlated with FOXM1 in TCGA HNSCC cohort and multiple independent GEO datasets including GSE127165, GSE30784, GSE37991, GSE40774, GSE41613, GSE65858, and GSE117973.

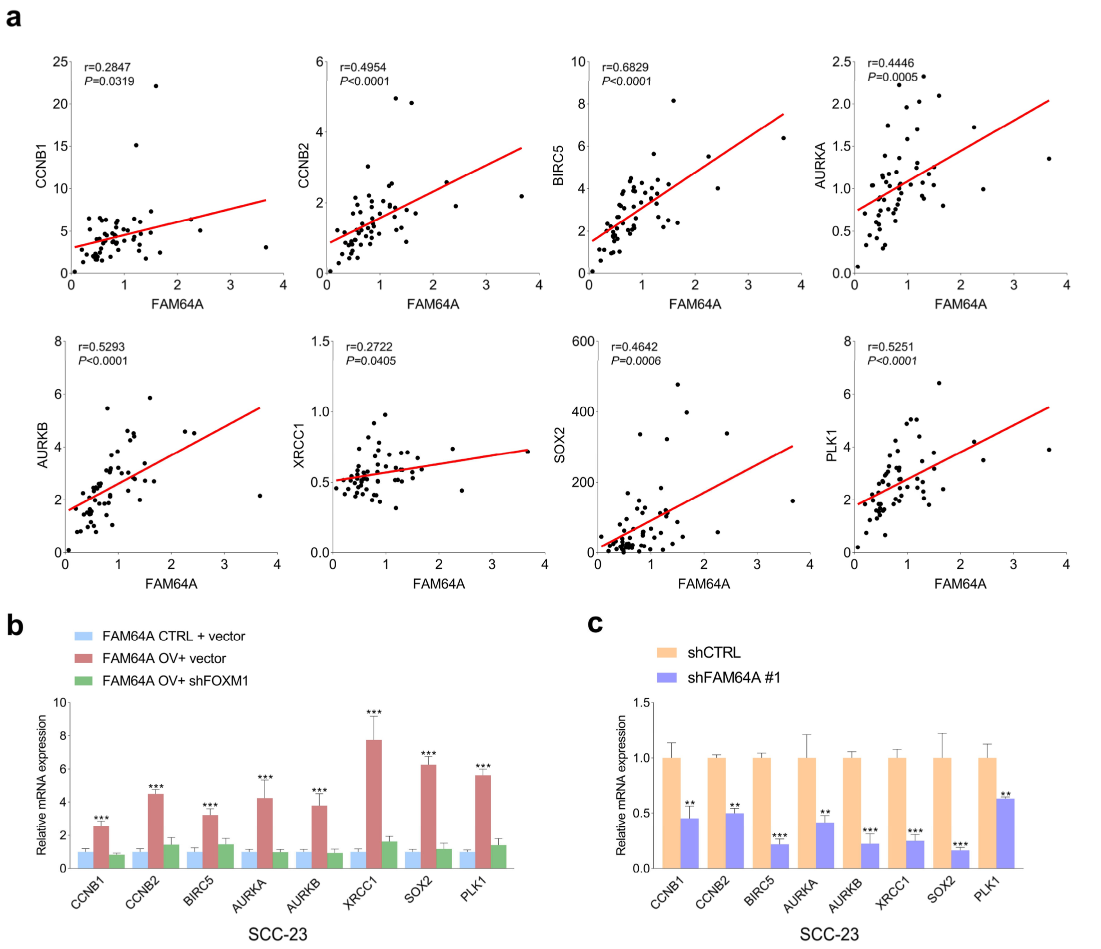

Figure S12 FAM64A enhanced the transcriptional activity of FOXM1. **a** FAM64A expression was positively associated with the levels of *CCNB1*, *CCNB2*, *BIRC5*, *AURKA*, *AURKB*, *XRCC1*, *SOX2* and *PLK1* in GSE127165. **b** FAM64A upregulation increased the expression of FOXM1 target genes in SCC-23 cells, and FOXM1 downregulation abrogated these enhancing effects. **c** FAM64A downregulation decreased the levels of FOXM1-target genes in SCC-23 cells. Data are presented as the mean ± SD. $^{**}P < 0.01$, $^{***}P < 0.001$.

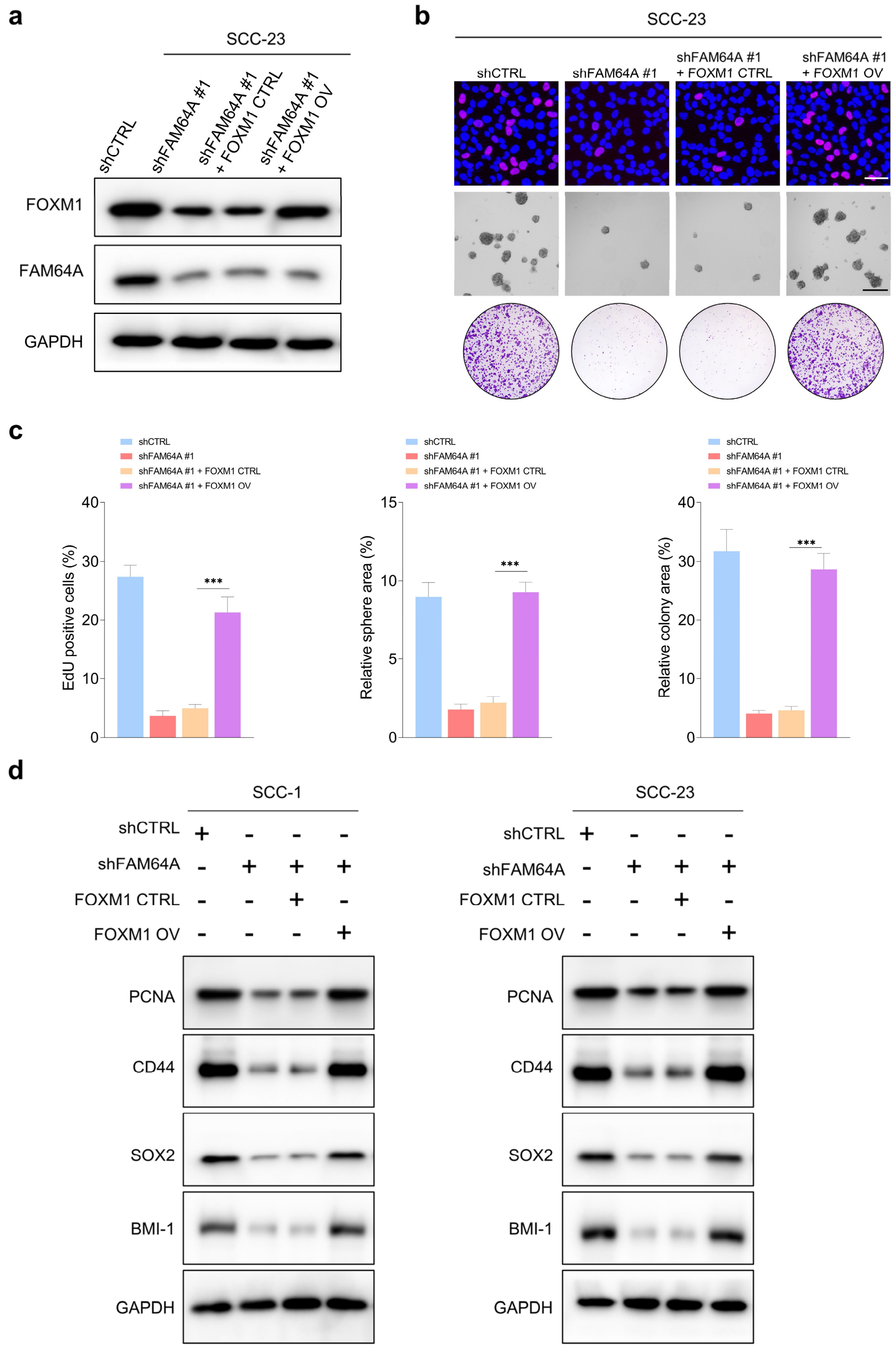

Figure S13 FOXM1 overexpression rescued the oncostatic effects of FAM64A depletion *in vitro*. **a** Western blot analysis of FOXM1 and FAM64A expression in SCC-23 cells with indicated modifications. **b, c** FAM64A depletion inhibited the proliferation, tumor sphere formation and colony formation capacities of SCC-23 cells, and these suppressive effects were reversed by ectopic expression of FOXM1. **d** FAM64A

depletion reduced the expression levels of PCNA, CD44, SOX2 and BMI-1 in HNSCC cell lines, and these repressive effectives were rescued by FOXM1 overexpression. Data are presented as the mean ± SD. *** $P < 0.001$. Scale bar: 50 μm for upper **(b)** and 200 μm for lower **(b)**.

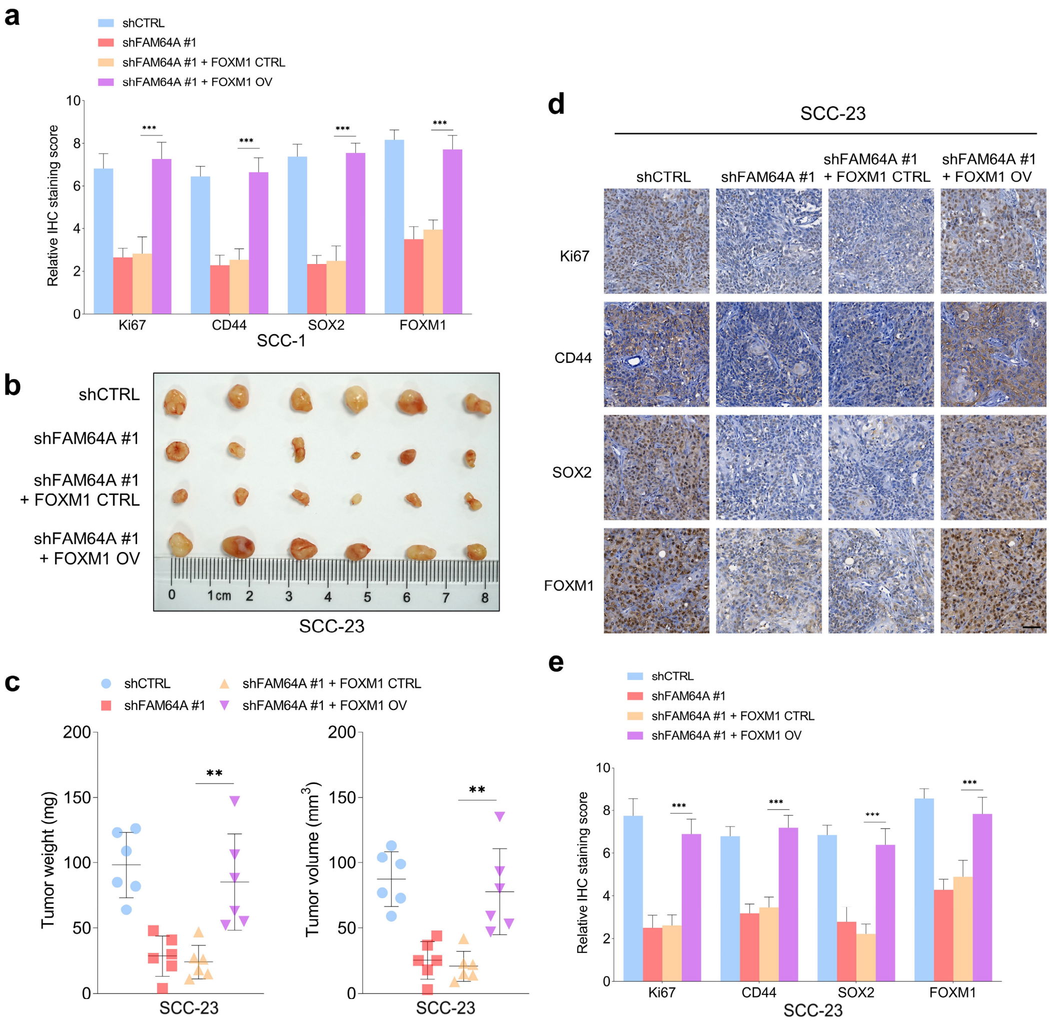

Figure S14 FOXM1 overexpression rescued the oncostatic effects of FAM64A depletion *in vivo.* **a** In SCC-1 cells, FAM64A depletion-induced reductions of Ki-67, SOX2, CD44 and FOXM1 in xenograft tumor tissues were abrogated by FOXM1 overexpression. **b-e** In SCC-23 cells, FAM64A depletion reduced tumor growth and the levels of Ki-67, SOX2, CD44 and FOXM1 in xenograft tumor tissues, and FOXM1 overexpression counteracted the inhibitory effects of FAM64A downregulation. Data are presented as the mean ± SD. **$P < 0.01$, *** $P < 0.001$. Scale bar: 50 μm.

Supplementary Table 1. The clinicopathological information of the HNSCC patients.

Clinicopathological parameters	Number of patients (n, %)
**Age**	
>=60	62 (55.36%)
<60	50 (44.64%)
**Gender**	
Male	83 (74.11%)
Female	29 (25.89%)
**Smoking status**	
Yes	56 (50.00%)
No	56 (50.00%)
**Alcohol drinker**	
Yes	41 (36.61%)
No	71 (63.39%)
**T stage**	
T1-T2	78 (69.64%)
T3-T4	34 (30.36%)
**Lymph node metastasis**	
No	69 (61.61%)
Yes	43 (38.39%)
**TNM stage**	
I-II	48 (42.86%)
III-IV	64 (57.14%)
**Differentiation**	
G1	59 (52.68%)
G2-G3	53 (47.32%)

Supplementary Table 2. Sequences of primers and oligos used in this study.

Gene	Sequence (5′-3′)
FOXM1	F: GGGCGCACGGCGGAAGATGAA
	R: CCACTCTTCCAAGGGAGGGCTC
CCNB1	F: CATGGTGCACTTTCCTCCTT
	R: AGGTAATGTTGTAGAGTTGGTGTCC
CCNB2	F: TGGAAAAGTTGGCTCCAAAG
	R: TCAGAAAAAGCTTGGCAGAGA
BIRC5	F: TGCCTGGCAGCCCTTTC
	R: CCTCCAAGAAGGGCCAGTTC
AURKA	F: GAGGTCCAAAACGTGTTCTCG
	R: ACAGGATGAGGTACACTGGTTG
AURKB	F: ATCAGCTGCGCAGAGAGATCGAAA
	R: CTGCTCGTCAAATGTGCAGCTCTT
XRCC1	F: GGGACCGGGTCAAAATTGTT
	R: ACCGTACAAAACTCAAGCCAAAG
SOX2	F: GAGCTTTGCAGGAAGTTTGC
	R: GCAAGAAGCCTCTCCTTGAA
PLK1	F: GACAAGTACGGCCTTGGGTA
	R: GTGCCGTCACGCTCTATGTA
GAPDH	F: TGCACCACCAACTGCTTAGC
	R: GGCATGGACTGTGGTCATGAG
shFAM64A #1	GCTCAGCTAAGAGTGCTTT
shFAM64A #2	TCCTGGAAACGCCTGGAAA
shFAM64A rescue	GCTCTGCGAAAAGCGCATT
